# Mid‐childhood autism sibling recurrence in infants with a family history of autism

**DOI:** 10.1002/aur.3182

**Published:** 2024-07-08

**Authors:** Tessel Bazelmans, Rowan Arthur, Greg Pasco, Elizabeth Shephard, Bosiljka Milosavljevic, Jannath Begum Ali, Andrew Pickles, Mark H. Johnson, Emily J. H. Jones, Tony Charman, The BASIS/STAARS Team, Jeni Baykoca, Anna Blasi, Patrick Bolton, Celeste Cheung, Kenny Chiu, Leila Dafner, Kim Davies, Mayada Elsabbagh, Janice Fernandes, Laurel Fish, Isobel Gammer, Teodora Gliga, Jeanne Guiraud, Rianne Haartsen, Sarah Kalwarowsky, Anna Kolesnik, Michelle Liew, Sarah Lloyd‐Fox, Helen Maris, Luke Mason, Mia Medas, Louise O'Hara, Laura Pirazzoli, Helena Ribeiro, Erica Salomone, Chloë Taylor, Leslie Tucker

**Affiliations:** ^1^ Institute of Psychiatry, Psychology & Neuroscience, King's College London London UK; ^2^ Faculdade de Medicina Universidade de São Paulo São Paulo Brazil; ^3^ School of Biological and Behavioural Sciences Queen Mary University of London London UK; ^4^ Centre for Brain and Cognitive Development, Birkbeck University of London London UK; ^5^ Department of Psychology University of Cambridge UK

**Keywords:** autism, diagnosis, family history, infants, recurrence likelihood, siblings

## Abstract

Autism sibling recurrence in prospective infant family history studies is ~20% at 3 years but systematic follow‐up to mid‐childhood is rare. In population and clinical cohorts autism is not recognized in some children until school‐age or later. One hundred and fifty‐nine infants with an older sibling with autism underwent research diagnostic assessments at 3 years and mid‐childhood (6 to 12 years (mean 9)). We report the autism sibling recurrence rate in mid‐childhood and compare developmental and behavioral profiles at mid‐childhood and 3 years in those with earlier versus later recognized autism, and those who had, or had not, received a community autism diagnosis. The autism recurrence rate in this sample in mid‐childhood was 37.1%, 95% CI [29.9%, 44.9%] and higher in boys than girls. Around half of those diagnosed with autism in mid‐childhood had not received a diagnosis at 3 years. Later, diagnosis was more common in girls than boys. While some had sub‐threshold symptoms at 3, in others late diagnosis followed a largely typical early presentation. Sibling recurrence based on community clinical diagnosis was 24.5%, 95% CI [18.4%, 31.9%]. Those who also had a community diagnosis tended to be older, have lower adaptive function and higher autism and inattention symptoms. Notwithstanding limitations of a single site study, modest sample size and limits to generalisability, autism sibling recurrence in family history infants may be higher in mid‐childhood than in studies reporting diagnostic outcome at 3 years. Findings have implications for families and clinical services, and for prospective family history studies.

## Lay Summary

Autism sibling recurrence in prospective infant family history studies is ~20% at 3 years but follow‐up to mid‐childhood is rare although in some children autism is not recognized until school‐age or later. We assessed 159 infants with an older sibling with autism at 3 years and in mid‐childhood (9 years). The autism sibling recurrence rate in mid‐childhood was 37.1%, 95% CI [29.9%, 44.9%] and higher in boys than girls. Only half of those diagnosed with autism in mid‐childhood had received a diagnosis at 3 years and later diagnosis was more common in girls than boys. Clinicians need provide ongoing monitoring of younger siblings of autism probands to be able to offer support to families when they express concern and seek services.

## INTRODUCTION

Prevalence estimates for autism in childhood are ~2% (Maenner et al., 2023; Roman‐Urrestarazu et al., 2021) and higher in males than females. In population studies the sibling recurrence rate is ~10% (Hansen et al., 2019; Jokiranta‐Olkoniemi et al., 2016; Sandin et al., 2014), reflecting the strong heritability of the condition (Sandin et al., 2014; Tick et al., 2016). In prospective studies of infants with a family history of autism, sibling recurrence at 3 year diagnostic evaluations is ~20% (Messinger et al., 2015; Ozonoff et al., 2011); and higher in males than females (Messinger et al., 2015; Ozonoff et al., 2011) and in siblings from multiplex (more than autistic sibling) than simplex families (McDonald et al., 2020; Ozonoff et al., 2011). Ascertainment, reporting and measurement biases might account for why recurrence estimates are higher than in population samples of older children. Higher recurrence may reflect characteristics of volunteer families recruited or recruitment of infants with pre‐existing delays or developmental concerns. However, studying later‐born siblings acts against reproductive stoppage, which might under‐estimate recurrence in population studies (Hoffmann et al., 2014) and direct clinical diagnostic assessments rather than reliance on parental report (Guo, 1998) might provide more accurate recurrence estimates. The early age of recruitment to infant sibling studies (commonly <12 months) might limit bias due to pre‐existing concerns or delays.

Most of what we know about sibling recurrence in prospective infant family history studies is restricted to diagnostic evaluations conducted at 3 years. However, there is good evidence that in some children autism might not be diagnosed in the early preschool period. DSM‐5 (American Psychiatric Association, 2022) states “symptoms must be present in early childhood but may not become fully manifest until social demands exceed limited capacities.” Population prevalence estimates are higher at 8 than 4 years (Christensen et al., 2019) and some children are diagnosed after 6 years despite having undergone earlier developmental evaluations (Davidovitch et al., 2015). In a clinically referred cohort seen for repeated autism diagnostic assessments at 2 and 9 years 11/135 (8%) of children given an autism diagnosis at 9 years by an expert group using standard diagnostic instruments had not been given a diagnosis at 2 (Lord et al., 2006).

Recent studies have followed autism family history infants to mid‐childhood. Shephard et al. (2017) reported on 42 siblings at age 7 years and 15 (35.7%) had autism. Whilst 10 had been diagnosed at 3 years, five were only later diagnosed and three children given a diagnosis at 3 years were not in mid‐childhood. Brian et al. (2016) followed 67 siblings to 9 years and 23 (34.3%) had autism, with six being only later diagnosed and one child no longer meeting diagnostic criteria. Landa et al. (2022) reported on 210 siblings at 6 years and 42 (20.0%) had autism but found that more children lost their diagnosis (*n* = 17) between 3 years and school‐age than gained a diagnosis (*n* = 6). Ozonoff et al. (2018) focused on 14 infant siblings^1^ only given a diagnosis in mid‐childhood after diagnostic assessments at 3 years and, whilst half had subthreshold clinical symptoms but were not given a diagnosis, half presented with no clear symptoms at 3 years (although some parents expressed concerns).

The current study reports mid‐childhood diagnostic outcomes and the sibling recurrence rate in mid‐childhood in an autism family history infant cohort first assessed before 12 months of age who also underwent 3‐year diagnostic assessments. We examine child and family factors related to recurrence and contrast recurrence in mid‐childhood with that from the earlier 3‐year assessments. We then utilize the systematic longitudinal cohort research design to investigate two additional issues:

(1) We expected to identify children with later diagnosed autism and contrast scores on standard developmental, autism and behavioral assessments at 3 years and mid‐childhood between the earlier versus later diagnosed children to identify factors related to later recognition.

(2) For children who had a research autism diagnosis we compare those with, and without, a local community clinical autism diagnosis to investigate factors that systematically influence community recognition compared to our prospective, repeated research diagnostic assessments.

## METHOD

### Participants

Two hundred and forty later‐born infant siblings (125 boys, 115 girls) were recruited in a prospective family history study.^2^ Age of first research assessment was younger than 12 months^3^ (mean (SD) = 7.26 (2.12)). The primary basis for recruitment was parent report of a community clinical diagnosis of autism in the older proband. In most cases in addition to this the proband also met the autism threshold on the Development and Well‐Being Assessment (DAWBA (Goodman et al., 2000)) and/or the Social Communication Questionnaire (SCQ (Rutter et al., 2003)) conducted as part of the current research study, and in many cases parents provided clinical reports for the research team to review (see Supplementary Material for details). This comprised three cohorts: Phase 1 (*n* = 50) reported in Shephard et al. (2017) and new Phase 2 (*n* = 116) and Phase 3 (*n* = 74) cohorts. Research assessments were undertaken at 5, 9 and 14 months and 2 and 3 years. Of 226 children retained at the 3 years, 163 took part in the mid‐childhood follow‐up at 6–12 years (*M* (SD) = 9.00 (1.31)) between July 2014 and September 2023.^4^ Four children who did not complete in‐person diagnostic assessment (parents completed questionnaires only) are excluded from the current analysis, leaving a sample of *n* = 159 siblings (80 boys, 79 girls).

All procedures involving human participants were approved by NHS RES London REC (14/LO/0170) and King's College London (RESCM‐18/19–10556). Parents provided written informed consent and children written/verbal informed assent appropriate to developmental level.

#### Family demographic characteristics

Ethnicity: Infant sibling ethnicity was characterized as Asian/Black African/Black Caribbean/Mixed versus White/European/Irish.

Family Sociodemographic Information: Annual household income was coded on a 5‐point ordinal scale (<£20,000, £20,000–£40,000, £40,000–£60,000, £60,000–£80,000, >£80,000). Maternal highest education level was coded on a 4‐point ordinal scale (16/GCSE, 18/School/College, Degree level, Postgraduate/Professional).

Simplex versus Multiplex Status: 41/240 (17.1%) infants had more than one older sibling (‘proband’) with an autism diagnosis and 10 parents^5^ had an autism diagnosis in addition to their older child. We base multiplex status (49/240 (20.4%)) on both older sibling and parent diagnosis, but results do not change when restricted to older sibling diagnosis only.

Proband characterization: As described under Participants, parent report of a community clinical diagnosis of autism in the older proband was the primary basis for recruitment. In addition, we measured oldest proband sex and SCQ score.

### Measures

#### Nine months and three years

Developmental ability: Early Learning Composite (ELC) from the Mullen Scales of Early Learning (Mullen, 1995) was used to measure of developmental ability. Vineland‐II Adaptive Behavior Composite (ABC) (Sparrow et al., 2005) was used to measure adaptive functioning.

Temperament: Parents completed the Infant Behavior Questionnaire‐Revised (IBQ‐R (Gartstein & Rothbart, 2003)) at 9 months and the Childhood Behavior Questionnaire (CBQ (Putnam & Rothbart, 2006)) at 3 years to measure the temperamental dimensions Surgency, Negative Affect, and Effortful Control.

#### Mid‐childhood

Cognitive ability and adaptive functioning: Wechsler abbreviated scale of intelligence–Second Edition (WASI‐II (Wechsler, 2011)) was used to assess full‐scale IQ (FSIQ). Vineland‐II (Phase 1) and Vineland‐3 Adaptive Behavior Composite (Sparrow et al., 2016) (Phase 2/3) was used to measure adaptive functioning.

#### Autism diagnostic and screening measures at 3 years and mid‐childhood

The standard observational and parent‐report diagnostic instruments autism diagnostic observation schedule‐2 (ADOS‐2) (Lord et al., 2012) and autism diagnostic interview‐revised (ADI‐R) (Lord et al., 1994) and parent‐report social communication questionnaire (SCQ) (Rutter et al., 2003) and social responsiveness scale‐2 (SRS‐2) (Constantino & Gruber, 2012) screening measures were completed.

#### ADHD and anxiety measures in mid‐childhood

Parent‐report Conners‐3 (Conners, 2008) was used to assess attention deficit hyperactivity disorder (ADHD) symptoms (T‐scores for the DSM‐IV‐TR Inattentive and Hyperactive/Impulsive scales). Parent‐report Spence Children's Anxiety Scale–Parent (SCAS‐P (Spence, 1999)) was used to assess anxiety symptoms (Total Anxiety T‐scores).

#### Research diagnostic assessments

At 3 years and mid‐childhood, a best estimate clinical diagnosis of autism spectrum disorder^6^ was made based on DSM‐5 criteria. This was informed by, but not dependent on, scores on the ADOS‐2, ADI‐R, SCQ, Vineland, and Mullen/WASI, researcher observations on the visit, and additional parent‐reported information, by experienced researchers with research reliability on the ADOS‐2 and overseen by a senior licensed clinical psychologist. Diagnosis in mid‐childhood involved review of all previous information, including 2‐ and 3‐year visits, and there was overlap in personnel involved, so decisions were not independent. For each diagnostic decision the assessing team discussed and agreed a global ordinal diagnostic confidence rating of ‘Low’, ‘Medium’, or ‘High’ certainty.

#### Community clinical diagnosis

At the mid‐childhood assessment parents reported whether or not the target infant sibling had received a community clinical diagnosis or not by local services.

### Data analysis

Data analysis was conducted in Stata 18 (StataCorp., 2023). Retention from recruitment, 9 months and 3 years to mid‐childhood and between group differences were examined univariately using chi‐squared tests for binary and ordinal variables, and *t*‐tests and one‐way ANOVAs with Tukey HSD post‐hocs, respectively, for two‐ and three‐group comparisons for interval variables. Multivariate logistic regression using Stata logit was used to determine unique predictors of retention, recurrence rate, earlier vs. later diagnosis, and concordance between community and research diagnosis. In these models, child (sex, age at mid‐childhood visit, ethnicity), family (maternal education, family income, simplex vs. multiplex status, maternal age, paternal age), proband (sex, SCQ score) and Phase were entered as predictors. The percentage of missing values ranged from zero to 14 (9%) and only 126 from 159 cases (79%) had full data under list wise deletion. We therefore used multiple imputation by chained equations (MICE) under the assumption that missing values are missing at random and included appropriate auxiliary variables for each model (see Supplementary Materials). The Stata mi impute chained command generated 50 imputed datasets with 10 burn‐in iterations. Imputed values were similar to observed values and results using list wise deletion similar to those using multiple imputation, so imputed results are presented. For relevant binary predictor variables odds ratios (OR) and 95% confidence intervals (CIs) are reported. Recurrence rates and 95% CIs were estimated using the Stata proportion command.

### Results

#### Retention

Retention from recruitment to mid‐childhood was 163/240 (67.9%). Retention was higher in Phase 1 (82.0%) than Phase 2 (66.4%), 𝜒^2^(1, *N* = 166) = 4.15, *p* < 0.05 and Phase 3 (60.8%) 𝜒^2^(1, *N* = 124) = 6.30, *p* < 0.05, and lower in Asian/Black/Mixed compared to White children (50.0% vs. 73.9%), 𝜒^2^(1, *N* = 232) = 10.68, *p* < 0.01 (Table 1). In the imputed multivariate regression Phase (Phase 1 > Phase 3, *t* = 2.37, *p* < 0.05), child ethnicity (*t* = 2.82, *p* < 0.01) and multiplex status (*t* = 2.10, *p* < 0.05; being higher in multiplex (78%) than simplex (65%) infants) were associated with retention but sibling sex, maternal and paternal age, maternal education, family income and proband sex and SCQ score were not (all *p* > 0.07) (see Table S9.1). Retention to mid‐childhood was not associated with 9‐month Mullen ELC, Vineland ABC or IBQ surgency, negative affect and effortful control in either univariate or the imputed multivariate model (all *p* > 0.20, see Table S2). Retention was not associated with 3‐year Mullen ELC, Vineland ABC, ADOS‐2, ADI‐R, SCQ, SRS or CBQ surgency, negative affect and effortful control, Vineland internalizing or externalizing scores or a research autism diagnosis in univariate models (all *p* > 0.12). In the imputed multivariate model those retained to mid‐childhood had nonsignificantly higher Vineland ABC (*p* = 0.08), lower ADOS‐2 (*p* = 0.08) and higher ADI‐R (*p* = 0.08) scores than those nonretained but no other variables approached significance (see Table S2 and Table S9.3).

**TABLE 1:** Sample characteristics of those seen versus not seen at mid‐childhood visit.

Recruitment	Seen in mid‐childhood						
	Seen MC (*N* = 163)			Not seen MC (*N* = 77)			
	Count	%	Row %	Count	%	Row %	χ^2^ (p)
Phase							6.39 (0.041)
1	41	25%	82%	9	12%	18%	
2	77	47%	66%	39	51%	34%	
3	45	28%	61%	29	38%	39%	
Child sex							1.16 (0.281)
Male	81	50%	65%	44	57%	35%	
Female	82	50%	71%	33	43%	29%	
Child ethnicity							10.68 (0.001)
Asian/African/Black/African Caribbean/Mixed	26	16%	50%	26	36%	50%	
White/European/Irish	133	84%	74%	47	64%	26%	
Annual household Income							3.85 (0.427)
Up to £20,000	12	8%	63%	7	12%	37%	
£20,000 to £40,000	42	27%	66%	22	37%	34%	
£40,000 to £60,000	42	27%	72%	16	27%	28%	
£60,000 to £80,000	23	15%	79%	6	10%	21%	
Above £80,000	34	22%	79%	9	15%	21%	
Maternal highest education							0.39 (0.941)
Up to 16/GCSE	14	9%	74%	5	7%	26%	
Up to 18/School/College	45	29%	67%	22	32%	33%	
Degree level	58	37%	71%	24	35%	29%	
Postgraduate/Professional	40	25%	70%	17	25%	30%	
Simplex versus multiplex status							2.62 (0.105)
Simplex	125	77%	65%	66	86%	35%	
Multiplex	38	23%	78%	11	14%	22%	
Proband sex							0.08 (0.773)
Male	143	88%	68%	68	89%	32%	
Female	19	12%	70%	8	11%	30%	

	*M*	(SD)	*N*	*M*	(SD)	*N*	*t*‐test (*p*)
Proband SCQ	23.61	(7.32)	148	23.10	(6.56)	62	−0.48 (0.635)

Abbreviations: MC, mid‐childhood; SCQ, Social Communication Questionnaire.

### Mid‐childhood recurrence

From 159 siblings seen in‐person in mid‐childhood 59 (*n* = 35 boys; *n* = 24 girls: sex ratio 1.46:1) met DSM‐5 criteria for autism, a recurrence rate of 37.1%, 95% CI [29.9%, 44.9%]. In univariate analysis, autism sibling recurrence was nonsignificantly higher in males 43.8%, 95% CI [33.3%, 54.8%] versus females 30.4%, 95% CI [21.2%, 41.4%], 𝜒^2^(1, *N* = 159) = 3.04, *p* = 0.08, but did not differ by Phase (Phase 1: 32.5%; Phase 2: 42.7%; Phase 3: 31.8%), multiplex status (simplex 35.3% vs. multiplex 43.2%), proband sex, child ethnicity or sibling age, family income, maternal education, or proband SCQ score, see Table S2 (all *p* > 0.21). In the imputed multivariate logistic regression only sibling sex was associated with recurrence rate (*t* = 1.97, *p* < 0.05) being higher in males than females, OR = 2.08, 95% CI [1.00, 4.30], all other *p* > 0.19 (see Table S9).

Twenty one of the 59 children (36%) with a research autism spectrum diagnosis were below the autism spectrum cut‐point on the ADOS‐2 and 16 (28%) on the ADI‐R but only 6 (10%) did not meet autism spectrum criteria on either instrument (Table S6). Sixteen (16%) nonautistic children scored above the autism spectrum threshold on the ADOS‐2 and five (5%) on the ADI‐R. Siblings with autism had higher scores on autism diagnostic and screening instruments (ADOS‐2, ADI‐R, SCQ, SRS) than siblings without autism (see Table 2). Those with autism also had higher scores on the Conners‐3 Inattentive and Hyperactive ADHD subscales and SCAS‐P anxiety measure and lower Vineland ABC scores but the groups did not differ on FSIQ.

**TABLE 2:** Mid‐childhood measures of symptoms by diagnostic outcome.

	Autism outcome in mid‐childhood						
	Autism (*N* = 59)			Not autism (*N* = 100)			
Male:Female *N* (%)	35 (59%):24 (41%)			45 (45%):55 (55%)			χ^2^ = 3.04 (*p* = 0.081)
	*M*	(SD)	*N*	*M*	(SD)	*N*	*t*‐test (*p*)
Child Age (months)	109.24	(15.50)	59	106.54	(15.05)	100	−1.08 (0.288)
WASI FSIQ	104.24	(18.02)	58	109.20	(14.82)	98	1.86 (0.064)
Vineland ABC	85.18	(14.51)	55	102.06	(12.36)	98	7.61 (<0.001)
ADOS‐2 SA CSS	5.73	(2.69)	59	2.87	(2.15)	98	−7.33 (<0.001)
ADOS‐2 RRB CSS	4.44	(2.86)	59	2.14	(2.16)	98	−5.7 (<0.001)
ADOS‐2 Total CSS	5.10	(2.70)	59	2.28	(1.82)	98	−7.84 (<0.001)
ADI‐R Social	12.97	(6.53)	58	2.63	(3.65)	99	−12.74 (<0.001)
ADI‐R Communication	10.59	(5.74)	58	2.31	(3.26)	99	−11.53 (<0.001)
ADI‐R RRB	3.36	(2.38)	58	0.66	(1.05)	99	−9.80 (<0.001)
ADI‐R Onset	2.47	(1.54)	58	0.61	(1.03)	99	−9.07 (<0.001)
SCQ Total mid	12.49	(7.70)	50	2.71	(3.64)	98	−10.5 (<0.001)
SRS T	72.12	(16.67)	50	49.26	(8.87)	93	−10.72 (<0.001)
SRS SCI T	72.14	(16.45)	50	49.38	(8.96)	94	−10.76 (<0.001)
SRS RRB T	69.18	(17.42)	50	48.99	(8.86)	94	−9.23 (<0.001)
Conners‐3 AN T	66.66	(16.78)	50	52.84	(12.59)	95	−5.58 (<0.001)
Conners‐3 AH T	68.46	(15.31)	50	54.00	(13.93)	95	−5.74 (<0.001)
SCAS‐P T	57.29	(9.92)	45	53.47	(8.57)	87	−2.30 (0.023)

Abbreviations: ABC, Vineland Adaptive Behavior Composite; ADOS‐2 CSS, Autism Diagnostic Observation Schedule‐2 Calibrated Severity Score, ADI‐R, Autism Diagnostic Interview‐Revised; AN, Inattentive; AH, Hyperactivity; FSIQ, Full scale IQ; RRB, Restricted interests and Repetitive Behavior; SCQ, Social Communication Questionnaire, SRS, Social Responsiveness Scale, SCI, Social Communication; SCAS‐P, Spence Children's Anxiety Scale–Parent, *T*, *t*‐scores; WASI, Wechsler Abbreviated Scale of Intelligence.

At 3 years, 38 of the 226 siblings (30 boys, 8 girls) met DSM‐5 criteria for autism spectrum disorder a recurrence rate of 16.8%, 95% CI [12.5%, 22.3%] and was higher in males 26.1%, 95% CIs [18.8%, 34.9%] than females 7.2%, 95% CIs [3.6%, 13.8%], *p* < 0.001. See Supplementary Material for details of retention to 3 years and predictors of 3‐year recurrence.

### Comparing children with an earlier versus a later diagnosis of autism

Of 59 siblings with an autism diagnosis in mid‐childhood 28 (47.5%) had received a diagnosis at the 3‐year assessment (‘earlier diagnosed’) but 31 (52.5%) had not (‘later diagnosed’). Three children who were given a research autism diagnosis at the 3‐year assessment were subsequently not given a research autism diagnosis at the mid‐childhood time point. Univariately, later diagnosis was nonsignificantly more common in girls (66.7% of girls with a mid‐childhood diagnosis) versus boys (42.9%), 𝜒^2^ (1, *N* = 59) = 3.24, (*p* = 0.07) but was not associated with Phase, multiplex status, child ethnicity, proband sex or SCQ score, maternal education, family income or maternal and paternal age (all *p* > 0.11). In the imputed multivariate logistic regression sibling sex was marginally associated with later vs. earlier diagnosis (*t* = 1.89, *p =* 0.06) being higher in females than males, OR = 4.36, 95% CI [0.95, 19.90], all other *p* > 0.08. Diagnostic certainty rating was higher in those with an earlier and stable diagnosis (7% low, 21% medium, 71% high) than those with a later diagnosis (23% low, 39% medium, 39% high), 𝜒^2^(1, *N* = 59) = 6.64, *p* < 0.05.

**FIGURE 1:**
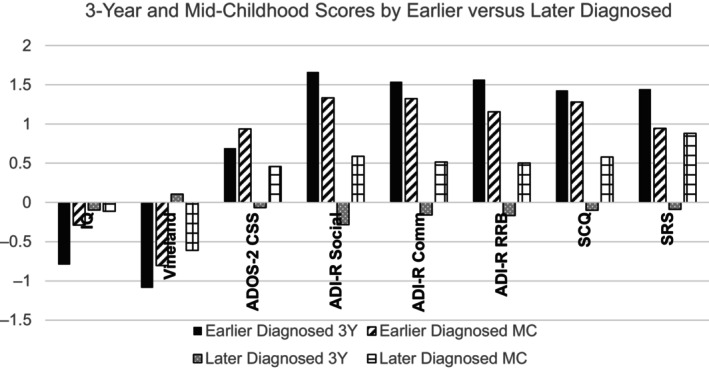
3‐year and mid‐childhood scores by earlier versus later diagnosed. To contrast scores of the Early versus Later diagnosed groups on the developmental and autism measures used at 3‐years and mid‐childhood z‐scores derived separately for each measure from the current sample at each timepoint are shown. IQ, Mullen ELC (3‐Year)/WASI FSIQ (Mid‐Childhood); ABC, Vineland Adaptive Behavior Composite; ADOS‐2 CSS, Autism Diagnostic Observation Schedule‐2 total Calibrated Severity Score, ADI‐R, Autism Diagnostic Interview‐Revised; Social, Social domain; Comm, Communication domain; RRB, Restricted and repetitive behaviors domain; SCQ, Social Communication Questionnaire, SRS, Social Responsiveness Scale *T*‐score.

Mid‐childhood and 3‐year profiles of the ‘Earlier’ (*n* = 28) and ‘Later’ diagnosed (*n* = 31) with the ‘Never diagnosed’ (*n* = 97) siblings are shown in Table 3 7 and Figure 1. In mid‐childhood the groups did not differ in age or FSIQ but the Earlier and Later diagnosed groups had lower adaptive behavior scores than the Never diagnosed group. The Earlier diagnosed group had higher scores on the ADOS‐2 Total CSS score, ADI‐R and SCQ than the Later diagnosed group who, in turn, had higher scores than the Never diagnosed group. The Earlier and Later diagnosed groups also had higher SRS and Conners‐3 Inattention and Hyperactivity scores than the Never diagnosed group but the three groups did not differ on SCAS‐P anxiety score. At 3 years the earlier diagnosed had lower Mullen ELC and Vineland ABC scores and higher ADOS, ADI, SCQ and SRS scores than both the later diagnosed and never diagnosed who did not differ from each other.

**TABLE 3:** Mid‐childhood and 3‐year scores by earlier versus later versus never diagnosed with autism.

	Earlier (3 year) versus later (mid‐childhood) diagnosis									
	Earlier diagnosed (*N* = 28)			Later diagnosed (*N* = 31)			Never autism (*N* = 97)			
Male:Female N (%)	20 (71%):8 (29%)			15 (48%):16 (52%)			42 (43%): 55 (57%)			*χ* ^2^ = 6.89 (*p* = 0.032)
	*M*	(SD)	*N*	*M*	(SD)	*N*	*M*	(SD)	*N*	ANOVA F (*p*)
**Mid‐childhood**										
Child Age (months)	106.14	(14.53)	28	112.03	(16.04)	31	107.02	(15.02)	97	1.51 (0.225)
WASI FSIQ	102.70	(18.15)	27	105.58	(18.09)	31	108.92	(14.73)	95	1.75 (0.177)
Vineland ABC	83.62^a^	(15.13)	26	86.59^a^	(14.04)	29	101.61^b^	(12.26)	95	27.58 (<0.001)
ADOS‐2 SA CSS	6.29^a^	(2.52)	28	5.23^a^	(2.78)	31	2.84^b^	(2.16)	95	28.69 (<0.001)
ADOS‐2 RRB CSS	4.46^a^	(3.14)	28	4.42^a^	(2.63)	31	2.11^b^	(2.11)	95	16.81 (<0.001)
ADOS‐2 Total CSS	5.75^a^	(2.73)	28	4.52^b^	(2.57)	31	2.25^c^	(1.82)	95	33.75 (<0.001)
ADI‐R Social	15.81^a^	(6.18)	27	10.48^b^	(5.85)	31	2.54^c^	(3.66)	96	99.52 (<0.001)
ADI‐R Communication	13.07^a^	(5.61)	27	8.42^b^	(4.99)	31	2.26^c^	(3.29)	96	82.11 (<0.001)
ADI‐R RRB	4.15^a^	(2.66)	27	2.68^b^	(1.90)	31	0.65^c^	(1.06)	96	56.50 (<0.001)
ADI‐R Onset	3.11^a^	(1.55)	27	1.90^b^	(1.30)	31	0.57^c^	(1.01)	96	53.91 (<0.001)
SCQ	15.07^a^	(6.54)	24	10.10^b^	(8.04)	26	2.69^c^	(3.69)	95	63.20 (<0.001)
SRS T	72.67^a^	(15.77)	24	71.62^a^	(17.77)	26	49.39^b^	(8.97)	90	54.81 (<0.001)
SRS SCI T	72.58^a^	(15.36)	24	71.73^a^	(17.70)	26	49.48^b^	(9.05)	91	55.42 (<0.001)
SRS RRB T	69.96^a^	(17.88)	24	68.46^a^	(17.30)	26	49.19^b^	(8.93)	91	40.52 (<0.001)
Conners‐3 AN T	66.00^a^	(16.27)	24	67.27^a^	(17.52)	26	53.13^b^	(12.69)	92	14.55 (<0.001)
Conners‐3 AH T	66.96^a^	(13.55)	24	69.85^a^	(16.92)	26	54.36^b^	(14.01)	92	15.56 (<0.001)
SCAS‐P T	58.45	(9.96)	22	56.17	(9.98)	23	53.32	(8.67)	84	3.12 (0.048)
**36 months**										
Mullen ELC	87.48^a^	(24.15)	27	103.03^b^	(22.30)	31	110.99^b^	(19.12)	96	13.79 (<0.001)
Vineland ABC	81.48^a^	(14.44)	27	96.70^b^	(11.09)	30	98.98^b^	(10.11)	95	26.03 (<0.001)
ADOS‐2 SA CSS	4.61^a^	(2.92)	28	3.23^b^	(2.46)	31	2.63^b^	(2.05)	97	8.04 (<0.001)
ADOS‐2 RRB CSS	5.75^a^	(2.20)	28	3.90^b^	(2.47)	31	3.77^b^	(2.45)	97	7.52 (<0.001)
ADOS‐2 Total CSS	4.21^a^	(3.05)	28	2.48^b^	(2.10)	31	2.09^b^	(1.77)	97	10.95 (<0.001)
ADI‐R Social	12.75^a^	(5.35)	28	2.52^b^	(2.85)	31	1.89^b^	(2.65)	97	119.54 (<0.001)
ADI‐R Communication	10.82^a^	(4.00)	28	3.00^b^	(3.19)	31	1.91^b^	(2.97)	97	84.25 (<0.001)
ADI‐R RRB	4.79^a^	(2.56)	28	1.03^b^	(1.49)	31	0.48^b^	(0.91)	97	95.95 (<0.001)
ADI‐R Onset	2.93^a^	(1.21)	28	0.81^b^	(1.14)	31	0.64^b^	(1.00)	97	51.06 (<0.001)
ADI‐R Toddler Algorithm	17.64^a^	(6.51)	28	3.52^b^	(3.64)	31	2.14^b^	(3.43)	97	152.44 (<0.001)
SCQ	16.00^a^	(6.66)	27	5.42^b^	(5.12)	29	3.39^b^	(4.65)	91	62.61 (<0.001)
SRS T	67.19^a^	(13.70)	26	48.11^b^	(8.82)	27	44.39^b^	(7.54)	88	62.02 (<0.001)

*Note*: Three children lost their diagnosis and are not included in this table. Groups marked with different superscript letters (a, b, c) differed significantly with Tukey's HSD correction applied (*p* < 0.05).

Abbreviations: ABC, Vineland Adaptive Behavior Composite; ADOS‐2 CSS, Autism Diagnostic Observation Schedule‐2 Calibrated Severity Score; ADI‐R, Autism Diagnostic Interview‐Revised; AN, Inattentive; AH, Hyperactivity; ELC, Mullen Early Learning Composite; FSIQ, Full scale IQ; RRB, Restricted interests and Repetitive Behavior; SCAS‐P, Spence Children's Anxiety Scale–Parent; SCI, Social Communication; SCQ, Social Communication Questionnaire; SRS, Social Responsiveness Scale; T, *t*‐scores; WASI, Wechsler Abbreviated Scale of Intelligence.

Individual profiles of scores and parent and researcher concerns noted at the 3‐year assessment of later diagnosed children are shown in Table S7. About half had low ADOS‐2, ADI‐R and SCQ scores and no parent concerns or researcher atypicalities noted; about half had above threshold scores on some, but commonly not all, of these measures or concerns or atypicalities were noted but they were not given a research autism diagnosis.

### Comparing research diagnosis to community clinical diagnosis

Thirty‐nine children (24 boys, 15 girls) had a community clinical diagnosis at the time of the mid‐childhood assessment. This included 37 of 59 (62.7%) children with a mid‐childhood research autism diagnosis and two children not given a research autism diagnosis.^8^ Sibling recurrence based on community clinical diagnosis was 24.5%, 95% CI [18.4%, 31.9%] and nonsignificantly higher in boys (30.0%) versus girls (19.0%) (*p* = 0.11) and in multiplex (35.1%) versus simplex (21.3%) siblings (*p* = 0.09). In the imputed multivariate model, sibling sex (OR = 2.39, 95% CI [1.03, 5.57], *t* = 2.02, *p* < 0.05) and marginally multiplex status (OR = 2.31, 95% CI [0.91, 5.88], *t* = 1.73, *p =* 0.08) were associated with a local community clinical diagnosis.

Mid‐childhood and 3‐year scores of siblings with a research autism diagnosis who did (*n* = 37) and did not (*n* = 22) also have a community clinical diagnosis are shown in Table 4. In univariate tests those who also had a community diagnosis had lower Vineland ABC scores and higher mid‐childhood ADI‐R, SCQ, and Conners ADHD Inattention scores than those who did not. In the imputed multivariate model SCQ score (*t* = 2.13, *p* < 0.05) and marginally sibling age (*t* = 1.89, *p =* 0.06) were higher in those who also had a community diagnosis (all other *p* > 0.31). Those with a community diagnosis had lower 3‐year Mullen ELC and adaptive scores and higher ADI‐R, SCQ and SRS scores in univariate tests than those without but only 3‐year SCQ score (*p* = 0.07) was marginally associated in the multivariate model (all other *p* > 0.34). Community clinical diagnosis was marginally higher in those with an earlier (75.0%) versus a later (51.6%) research diagnosis, 𝜒^2^(1, *N* = 59) = 3.44, *p* = 0.064.

**TABLE 4:** Mid‐childhood and 3‐year scores of children with a research diagnosis with and without a community diagnosis of autism.

	Community diagnosis of autism						
	Research and community (*N* = 37)			Research only (*N* = 22)			
Child sex (Male: Female)	23 (62%): 14 (38%)			12 (55%): 10 (45%)			*χ* ^*2*^ = 0.33 (*p* = 0.565)
	*M*	(SD)	*N*	*M*	(SD)	*N*	*t*‐test (*p*)
**Mid‐childhood**							
Child Age (months)	111.27	(13.85)	37	105.82	(17.75)	22	−1.31 (0.194)
WASI FSIQ	101.44	(18.41)	36	108.82	(16.77)	22	1.53 (0.132)
Vineland ABC	81.64	(13.22)	33	90.50	(15.02)	22	2.31 (0.025)
ADOS‐2 SA CSS	5.95	(2.47)	37	5.36	(3.05)	22	−0.8 (0.426)
ADOS‐2 RRB CSS	4.24	(2.99)	37	4.77	(2.67)	22	0.68 (0.496)
ADOS‐2 Total CSS	5.27	(2.52)	37	4.82	(3.00)	22	−0.62 (0.538)
ADI‐R Social	15.36	(5.97)	36	9.05	(5.52)	22	−4.02 (<0.001)
ADI‐R Communication	12.22	(5.63)	36	7.91	(4.95)	22	−2.96 (0.004)
ADI‐R RRB	3.89	(2.48)	36	2.50	(1.97)	22	−2.23 (0.030)
ADI‐R Onset	3.03	(1.28)	36	1.55	(1.50)	22	−4.01 (<0.001)
SCQ	15.29	(6.00)	29	8.61	(8.24)	21	−3.32 (0.002)
SRS T	75.48	(14.28)	29	67.48	(18.88)	21	−1.71 (0.094)
Conners‐3 AN T	70.66	(16.90)	29	61.14	(15.32)	21	−2.04 (0.047)
Conners‐3 AH T	71.38	(13.46)	29	64.43	(17.06)	21	−1.61 (0.114)
SCAS‐P T	59.12	(9.37)	26	54.79	(10.36)	19	−1.46 (0.151)
**3 years**							
Mullen ELC	90.42	(25.61)	36	104.59	(19.36)	22	2.23 (0.030)
Vineland ABC	85.51	(14.94)	35	95.82	(12.45)	22	2.70 (0.009)
ADOS‐2 SA CSS	3.97	(2.67)	37	3.73	(2.95)	22	−0.33 (0.744)
ADOS‐2 RRB CSS	4.97	(2.60)	37	4.45	(2.36)	22	−0.77 (0.447)
ADOS‐2 Total CSS	3.46	(2.77)	37	3.05	(2.65)	22	−0.56 (0.575)
ADI‐R Social	8.70	(6.76)	37	5.14	(5.92)	22	−2.05 (0.045)
ADI‐R Communication	8.22	(5.36)	37	4.18	(4.25)	22	−3.01 (0.004)
ADI‐R RRB	3.41	(2.88)	37	1.82	(2.36)	22	−2.18 (0.033)
ADI‐R Onset	2.16	(1.54)	37	1.23	(1.51)	22	−2.27 (0.027)
ADI‐R Toddler Algorithm	12.43	(9.00)	37	6.50	(7.16)	22	−2.63 (0.011)
SCQ	13.12	(8.14)	35	6.20	(5.36)	21	−3.46 (0.001)
SRS T	61.13	(15.48)	31	52.32	(12.64)	22	−2.21 (0.032)

Abbreviations: ABC, Vineland Adaptive Behavior Composite; ADOS‐2 CSS, Autism Diagnostic Observation Schedule‐2 Calibrated Severity Score; ADI‐R, Autism Diagnostic Interview‐Revised; ELC, Mullen Early Learning Composite; FSIQ, Full Scale IQ; SCQ, Social Communication Questionnaire; SRS, Social Responsiveness Scale; AN, Inattentive; AH, Hyperactivity; SCAS‐P, Spence Children's Anxiety Scale–Parent; T, *t*‐scores; WASI, Wechsler Abbreviated Scale of Intelligence.

## DISCUSSION

### Sibling recurrence rate

The mid‐childhood sibling autism recurrence rate of 37.1% in family history infants is higher than in studies reporting recurrence (~20%) at 3‐year diagnostic evaluations (Messinger et al., 2015; Ozonoff et al., 2011). However, it is similar to a previous study of infants followed to mid‐childhood by Brian et al. (2016) (34.3%), although Landa et al. (2022) found a lower recurrence rate (20%) at age 6 years. Recurrence was higher in males than females, in line with previous family history and population studies (Maenner et al., 2023; Ozonoff et al., 2011; Roman‐Urrestarazu et al., 2021). At 3‐year diagnostic assessments, the recurrence rate (16.8%) was similar to multisite studies (Messinger et al., 2015; Ozonoff et al., 2011). Ozonoff et al. (2011) reporting sibling recurrence of 18.7% at age 3 years noted “outcome was determined before the age that milder forms of ASD, including Asperger disorder, are accurately diagnosed, the true recurrence rate may in fact be higher than reported here” (p. e492) and the recurrence rate we found in mid‐childhood suggests this may be the case. Mid‐childhood sibling recurrence based on community clinical diagnosis was 24.5%.

This high recurrence rate cannot be generalized outside the current moderate‐size, single‐site sample and requires replication in other cohorts and in large, multi‐site studies to establish a reliable estimate for mid‐childhood recurrence in family history infant siblings, as has been done for 3‐year outcomes (Messinger et al., 2015; Ozonoff et al., 2011). However, it is important to consider factors that might explain the recurrence rate we found and whether it relates to the wider increase seen in autism prevalence estimates over the past few decades (Fombonne, 2018).

Our sample had a higher rate of multiplex siblings (37 of 159 (23.3%) in mid‐childhood) than in studies reporting recurrence at 3‐years (7.8% in (Messinger et al., 2015); 6.0% in (Ozonoff et al., 2011)), indicating a higher familial loading. However, unlike previous studies (McDonald et al., 2020; Ozonoff et al., 2011), multiplex status was not significantly associated with recurrence either in mid‐childhood or at 3 years, although it was marginally associated with community clinical diagnosis. There is concern about over‐diagnosis of autism due to reliance on scores on diagnostic instruments and the absence of an expert formulation‐based application of the diagnostic criteria (Bishop & Lord, 2023; Fombonne, 2023) but this is not the case in the present study. We employed a consensus ‘best estimate’ DSM‐5 clinical diagnostic process informed by, but not dependent on, scores on the ADOS‐2 and ADI‐R, researcher observations, and additional parent‐reported information, by experienced researchers. One difference from BSRC consortium studies reporting recurrence at 3 years (Messinger et al., 2015; Ozonoff et al., 2011) is that they require the child to meet the ADOS‐2 autism spectrum disorder threshold in addition to clinical DSM‐5 criteria. We chose to prioritize the clinical consensus application of DSM‐5 diagnostic criteria in line with best practice recommendations (Bishop & Lord, 2023; Fombonne, 2023). One‐quarter to one‐third of children with a research autism diagnosis fell below the autism spectrum threshold on the ADOS‐2 or ADI‐R, respectively, but 90% were above threshold on one or both instruments. Both instruments have lower sensitivity in children with higher IQ (Havdahl et al., 2016), as do the current sample, and the ADI‐R reduced sensitivity when parents have not previously expressed concern about possible autism, which was true for some parents in this study (see below) (Havdahl et al., 2017).

One limitation on the generalisability of the recurrence rate we report is the representativeness of the sample. This goes beyond factors that affected retention, although White ethnicity was associated with higher retention indicating some selection bias. More generally, the sample sociodemographic characteristics (see Table 1) indicate that on average families had high income and parental education, although these were not associated with recurrence. There may be other parental or family characteristics that motivate families to volunteer for family history research studies and that relate to recurrence likelihood that we have not captured in the measures we used.

### Earlier versus later diagnosed children

Notably, 53% of those with a mid‐childhood autism diagnosis were ‘later diagnosed’ despite having undergone a research diagnostic assessment at 3 years. We and others have reported later identified cases in prospective infant studies (Brian et al., 2016; Grzadzinski et al., 2023; Ozonoff et al., 2018; Shephard et al., 2017). Earlier diagnosed children had higher scores on autism measures than the later diagnosed children in mid‐childhood but did not differ in IQ or adaptive behavior. At 3 years, later identified children had higher IQ and adaptive scores than the earlier identified children and their scores on autism measures did not differ from the ‘never diagnosed’ group. This is in line with previous findings that despite adequate sensitivity and specificity not all preschool autistic children are identified by the standard diagnostic assessments (ADI‐R, ADOS‐2) (Randall et al., 2018). Later diagnosis was more common in girls than boys in line with Burrows et al. (2023) and population and clinical studies (Lai & Szatmari, 2020). Half of the later identified children had sub‐threshold symptoms at 3 years or parents or researchers expressed some concerns but in half late emergence followed a more typical early presentation, as previously reported by Ozonoff et al. (2018). DSM‐5 allows for later‐emerging or later‐recognized symptoms and later diagnosis is found in clinical samples despite having undergone earlier assessments (Davidovitch et al., 2015; Lord et al., 2006). In some later diagnosed children, parents indicated that school entry and the need to navigate complex out‐of‐home social situations led to difficulties becoming more apparent, in line with DSM‐5 (“until social demands exceed limited capacities”).

Three children given an autism diagnosis at 3 years were not considered to have autism at mid‐childhood. Two showed sub‐threshold autism traits and a third was considered by the research team and parents to be typically developing, and whilst parents had reported concerns at age 3, they had no current concerns. Brian et al. (2016) reported one child who no longer met criteria for a research diagnosis in mid‐childhood but they continued to have a clinical diagnosis and on review was still considered to have autism. Two studies (Grzadzinski et al., 2023; Landa et al., 2022) have reported a larger number of cases ‘losing diagnosis’, although Grzadzinski et al. (2023) noted many continued to have sub‐threshold autism traits. Both Landa et al. (2022) and Grzadzinski et al. (2023) conducted initial diagnostic evaluations before age 3 years (15 months and 24 months, respectively) and this might in part account for a higher proportion of children moving from autism to nonautism diagnostic status.

### Research versus community diagnosis

Sixty three percent of those with a research diagnosis had a local community clinical diagnosis. Those with a community diagnosis were older, had lower adaptive behavior and higher scores on parent‐informed autism and ADHD measures than those without. Higher levels of autism severity and co‐occurring ADHD and lower adaptive function may explain why these children had been assessed and diagnosed by local services. However, ~40% of children with a research diagnosis did not have a community diagnosis. Some children were awaiting assessment, others had been assessed and did not receive a diagnosis, and in others whilst parents recognized autistic traits some were not concerned, did not think pursuing a formal diagnosis would benefit their child, or other medical, behavioral, or mental health issues were more pressing. In the UK, wait times for autism assessments are long (NHS England, 2023) and not all families had been able to access services. In line with this, in a population‐representative UK cohort only 21% of autistic children were diagnosed by age 5 years, 51% between 5 and 11 years, and 28% after age 11 years (Hosozawa et al., 2020).

### Limitations

We report a single site study with a modest sample size. Across studies there is variability in recurrence rates. The BSRC consortium papers reporting recurrence at 3 years note site effect but rates by site are not reported (Messinger et al., 2015; Ozonoff et al., 2011). Individual studies have reported 3‐year recurrence rates ranging from 27.5% (Zwaigenbaum et al., 2021) to 14% (Yoder et al., 2009) and we found variability in mid‐childhood recurrence across the three Phases from 32% to 43%. Retention from recruitment to mid‐childhood was only moderate (68%). There was some evidence of differential attrition by background characteristics (nonWhite ethnicity), although no evidence of more, or less, affected siblings being retained from 9 months or 3 years. However, our use of multiple imputation with auxiliary variables in the statistical analysis accounted for some of the potential bias from sample attrition and missing data. We did not include a quantitative measure of parent (or researcher) concerns at the infant or toddler assessments to assess whether this impacted retention, as has been done in some previous infant sibling studies (Ozonoff et al., 2023; Sacrey et al., 2015).

We did not directly assess older probands and the inclusion criteria for the sample was parent‐report of a community clinical diagnosis of autism but in most cases this was supported by scores on the DAWBA, SCQ and clinical reports. We did not systematically collect genetic data on older probands, parents and infant siblings, which would further inform our understanding of recurrence (Yoon et al., 2021). We ascertained whether the older proband (or parent) had a community clinical diagnosis of ADHD in addition to their diagnosis of autism^9^ but our sample is not adequately powered to assess whether other neuropsychiatric and neurodevelopmental conditions in family members affects autism recurrence.

Mid‐childhood diagnosis was not blind to 3‐year diagnosis and there was overlap in the research team involved, although discrepancy between the two timepoints suggests they were not yoked. We conducted no independent reliability of diagnostic decisions. During the period of the Covid‐19, pandemic adjustments were made to testing (mask wearing, Perspex screen), including for ADOS‐2 assessments for some children in our Phase 2 cohort (see Supplementary Material). Although, this is a non‐standard implementation of the ADOS‐2 assessment since the scores and information was used qualitatively and not categorically to inform diagnostic decisions the ADOS‐2 was scored in the usual way. Although later diagnosis was nominally higher in Phase 2 than Phase 1 and Phase 3 (see Table S5) this was not significant (both *p* > 0.14).

## CONCLUSIONS

If the sibling recurrence rate in prospective family history infant studies lies somewhere between the ~20% from multi‐site studies of 3‐year outcomes (Messinger et al., 2015; Ozonoff et al., 2011), the ~25% recurrence rate based on mid‐childhood community diagnosis and the ~ 35% recurrence rate based on mid‐childhood research diagnosis we report, it has implications for families and clinicians. Individual recurrence likelihood will depend on a number of genetic, familial and environmental factors, not all of which will be known (Marrus et al., 2021). In our study children who were only given an autism research diagnosis at the mid‐childhood but not at the 3‐year assessment were more likely to be girls and have higher IQ at the earlier assessment. Parents indicated that entry into school and other social challenges as their child developed made autism characteristics more apparent. This was consistent with scores on the ADI‐R and ADOS‐2 across these two timepoints (see Table 3). Thus, in the later diagnosed children both parent report and researcher observation identified more autism symptoms as children encountered more complex social environments in everyday life and social presses in the research assessment. It is important for parents and carers to know that in younger infant siblings an autism presentation may not always be clear in the preschool years. This will enable them to monitor and recognize the expression of autistic characteristics if they emerge later in development and then to seek advice and support. However, some parents of children who did not have a community autism diagnosis recognized behavioral difficulties or autism traits but were not concerned about their development or functioning and some families had accommodated to having multiple family members (one or more child, parents themselves, other relatives) with autism or autistic traits and saw this as a positive part of their family identity. Clinicians need to provide ongoing monitoring of younger siblings of autism probands to be able to offer support to families when they express concern and seek services.

Prospective infant studies should recognize that diagnostic outcome status based on 3‐year assessments are interim only and will change over time. One possibility is that previous findings indicating group level family history (‘elevated likelihood’) rather than 3‐year autism diagnostic outcome differences (Chawarska et al., 2016; Elsabbagh et al., 2009) might in part be driven by infant siblings who will go on to have a later‐emerging autism presentation and diagnosis. Experimental assays can test whether there are infant, preschool, or mid‐childhood neurodevelopmental differences between the earlier and later‐identified children to determine whether these might constitute ‘true’ subgroups or merely reflect differential trajectories and symptom expression (or recognition) of the same underlying condition (see (Bedford et al., 2017)). This may lead to reconsideration of prior interpretations and reanalysis of previous findings to incorporate the fundamental developmental nature both of autism and of prospective family history studies.

## AUTHOR CONTRIBUTIONS

Conceptualization: Tony Charman, Mark H. Johnson, Emily J. H. Jones, Andrew Pickles, Tessel Bazelmans, Elizabeth Shephard. Investigation: Tessel Bazelmans, Rowan Arthur, Greg Pasco, Elizabeth Shephard, Bosiljka Milosavljevic, Jannath Begum Ali. Data curation: Tessel Bazelmans, Greg Pasco, Elizabeth Shephard, Jannath Begum Ali. Formal analysis: Charman, Tessel Bazelmans. Funding Acquisition: Charman, Andrew Pickles, Mark H. Johnson, Emily J. H. Jones. Writing Original Draft: Charman. Writing Review and Editing: Tessel Bazelmans, Rowan Arthur, Greg Pasco, Elizabeth Shephard, Bosiljka Milosavljevic, Jannath Begum Ali, Andrew Pickles, Mark H. Johnson, Emily J. H. Jones. Approval of manuscript for submission: Tessel Bazelmans, Rowan Arthur, Greg Pasco, Elizabeth Shephard, Bosiljka Milosavljevic, Jannath Begum Ali, Andrew Pickles, Mark H. Johnson, Emily J. H. Jones, Charman.

## FUNDING INFORMATION

This research was supported by awards from the Medical Research Council (MR/R011427/1, G0701484, MR/K021389/1, MR/T003057/1), BASIS funding consortium led by Autistica, Autism Speaks. The results leading to this publication have received funding from the Innovative Medicines Initiative Joint Undertaking under grant agreement No 777394 for the project AIMS‐2‐TRIALS. This Joint Undertaking receives support from the European Union's Horizon 2020 research and innovation programme and EFPIA and AUTISM SPEAKS, Autistica, SFARI. Any views expressed are those of the authors and not necessarily those of the funders (IHI‐JU2). European Union Horizon Europe grant no. 101057385 (R2D2‐MH) and UK Research and Innovation (UKRI) under the UK government's Horizon Europe funding guarantee [grant no.10039383] as part of the Horizon Europe under grant agreement no. 101057385. Views and opinions expressed are however those of the authors only and do not necessarily reflect those of the European Union. Neither the European Union nor the granting authority can be held responsible for them. Capital equipment funding from the Maudsley Charity (980) and Guy's and St. Thomas’ Charity (STR130505). A. P. acknowledges support from the National Institute for Health Research (NIHR) through NF‐SI‐0617‐10120 and the NIHR Maudsley Biomedical Research Centre at South London and Maudsley NHS Foundation Trust and King's College London. The views expressed are those of the authors and not necessarily those of the NHS, the NIHR or the Department of Health and Social Care. The funders had no role in the design of the study; in the collection, analyses, or interpretation of data; in the writing of the manuscript; or in the decision to publish the results.

## CONFLICT OF INTEREST STATEMENT

T. C. has served as a paid consultant to F. Hoffmann‐La Roche Ltd. and Servier and receives royalties from Sage Publications and Guilford Publications. A. P. receives royalties from Western Psychological Services, Imperial College Press, and OUP. M. H. J. receives royalties from Wiley‐Blackwell, OUP and MIT Press. The remaining authors have declared that they have no competing or potential conflicts of interest.

## ETHICS STATEMENT

Ethical approval was obtained from the NHS National Research Ethics Service (NHS RES London REC 14/LO/0170) and Psychiatry, Nursing and Midwifery Research Ethics Subcommittee, King's College London (RESCM‐18/19–10,556).

## Supporting information


**Data S1.** Supporting Information.

## Data Availability

Data available following a review of requests as indicated here: https://www.basisnetwork.org/collaboration-and-project-affiliation/index.html.

## Footnotes

^1^
Including the 11 from Shephard et al. (2017) and Brian et al. (2016) above.

^2^
Two infants had a parent with an autism diagnosis and not a sibling. Results do not change when restricted to family history infant siblings with a proband (older sibling) with an autism diagnosis only.

^3^
One child's first research visit was after 12 months (14 months).

^4^
See Supplementary Material for adjustments to testing in Phase 2 during the Covid‐19 pandemic.

^5^
In two families both mother and father so *n* = 8 families.

^6^
In Phase 1 cohort 3‐year diagnostic assessments were completed using DSM‐IV‐TR but on subsequent review all cases who met criteria for a DSM‐IV pervasive developmental disorder were also considered to meet DSM‐5 criteria for autism spectrum disorder.

^7^
Excluding the n = 3 lost diagnosis cases since group too small to analyze.

^8^
For one child parents disagreed with the community diagnosis although were concerned about possible ADHD and anxiety; in the other parents reported high levels of autism symptoms but the research team considered symptoms overall were sub‐threshold.

^9^

*n* = 58 probands with autism also had a community clinical diagnosis of ADHD.
