# Supplementary material for: Mid‐childhood autism sibling recurrence in infants with a family history of autism
Source: Autism Res. 2024 Jul 8;17(7):1501–14. doi: 10.1002/aur.3182 (PMC11801122; doi:10.1002/aur.3182)
Supplement: Supplementary file 1 — Data S1. Supporting Information. [file AUR-17-1501-s001.docx]

**Mid-Childhood Autism Sibling Recurrence in Infants with a Family History of Autism**

**Supplementary Materials**

05-05-2024

**Additional Details re. Sample Recruitment and Characterization**

Participants were 240 later-born siblings recruited during infancy as part of a prospective family history study. Participants had an older sibling (proband) with a parent-reported community clinical autism diagnosis, confirmed using information from the Development and Well-Being Assessment (DAWBA) and the Social Communication Questionnaire (SCQ) by expert clinicians (TC, PB). Note: Two infants had a parent with an autism diagnosis and not a sibling with an autism diagnosis. As noted in the main paper, results do not change when restricted to family history infant siblings with a proband (older sibling) with an autism diagnosis only. 159 probands were positive on both the DAWBA and SCQ, 21 on DAWBA only (of which 9 had missing SCQ scores, 12 scored SCQ < 15), 37 on SCQ only (all DAWBA scores missing). For 23 children both the DAWBA and SCQ were missing and participation was based on parent report of local diagnosis only (though many provided diagnostic reports for the research team to review). 229 children were born full-term (37-42 weeks), 11 children were preterm (32-36 weeks). Infants were *M (SD)* 7.26 (2.12) months old (range 3 to 14 months) at their first infant research assessment. 49 multiplex families, 41 families had more than one sibling with a diagnosis (N=31 with 2 siblings, N=2 with 3 siblings, N=2 with 6 siblings (these participants were twins, N=5 with 2 siblings and a parent and N =1 with 2 siblings and 2 parents). Eight families had 1 sibling and 1 (N=6) or 2 (N=2) parents with a diagnosis. Parent-reported family medical histories were examined for significant conditions in the proband or extended family members (e.g., Fragile X syndrome, tuberous sclerosis) with no such conditions reported (see Supplementary Materials for details on sample characterisation).

**Note on Adjustments to Testing During Covid-19 Pandemic**

Phase 1 mid-childhood visits were conducted between July 2014 and August 2015. Phase 2 visits between May 2019 and July 2022. Phase 3 visits between August 2022 and September 2023. Phase 2 visits did not take place between March 2020 and May 2021 due to the Covid-19 pandemic and closure of the laboratory. Due to social distancing and infection control measures between June 2021 and June 2022 visits ADOS-2 assessments were conducted using face masks and a Perspex screen between the researcher and the child (*n* = 24) and face masks only (*n* = 10). Although this is a non-standard implementation of the ADOS-2 assessment since the scores and information was used qualitatively and not categorically to inform diagnostic decisions the ADOS-2 was scored in the usual way in order to report ADOS CSS scores for all children seen for a diagnostic assessment. Although Later diagnosis was nominally higher in Phase 2 than Phase 1 and Phase 3 (see Table S5) this was not significant (both *p* > .14).

**Details of the IBQ and CBQ versions used**

In all Phases the long form of the Infant Behavior Questionnaire-Revised (IBQ-R) was used at 8 months and the very short form (Phase 1 and 2) and short form (Phase 3) of the Childhood Behavior Questionnaire-short version (CBQ) at 3 years. For each scale, participants were included in the analysis if they had at least 60% of the items for that scale.

**Details of the WASI-II versions used**

Wechsler Abbreviated Scale of Intelligence – Second Edition (WASI-II (Wechsler, 2011)) full-scale IQ (FSIQ) was computed from the 4-subtest version (Block design, Vocabulary, Matrix Reasoning, Similarities) in Phase 1 and 2-subtest (Vocabulary, Matrix Reasoning) version in Phases 2 and 3.

**Auxiliary variables used in imputed multivariate models**

We used multiple imputation by chained equations (MICE) under the assumption that missing values are missing at random. The Stata mi impute chained command generated 50 imputed datasets with 10 burn-in iterations. Imputed values were similar to observed values and results using listwise deletion are similar to MI, so imputed results are presented.

Following recommendations for the inclusion of auxiliary variables in MI (Cornish et al., 2017; Lee et al., 2016) we included appropriate auxiliary variables for each model, as follows:

For (i) Retention from recruitment to mid-childhood; (ii) Retention from recruitment to 3 years; (ii) Mid-childhood recurrence likelihood; (iv) 3-year recurrence likelihood; (v) Earlier vs. later diagnosis; and (vi) Research vs. community diagnosis by background characteristics we included paternal education and number of siblings as auxiliary variables in addition to the predictor background variables: Phase, infant sex, child ethnicity, proband sex and SCQ score, multiplex status, maternal education, family income, maternal age and paternal age. Overall, paternal education and number of siblings were weakly correlated with the outcome variables but did correlate *r* = ~.30 to ~.40 with predictor variables with missingness e.g. maternal education, family income, maternal and paternal age.

For (i) Retention from 9 months to mid-childhood; (iii) Retention from 3 years to mid-childhood; (iii) Research vs. community diagnosis by mid-childhood characteristics; and (iv) Research vs. community diagnosis by 3-year characteristics we included Phase, infant sex, child ethnicity, proband sex and SCQ score, multiplex status, maternal education, family income, maternal age, paternal age as auxiliary variables in addition to the main (different) predictor variables for each model. These auxiliary variables were weakly correlated with the outcome variables and also with missingness (most *r* < .20).

For the key analyses (Retention to mid-childhood and Recurrence likelihood in mid-childhood) we conducted additional sensitivity analysis also including 3-year clinical scores (Mullen ELC, Vineland ABC, ADOS-2 CSS, ADI-R Toddler total, SCQ total, CBQ Surgency, Negative Affect and Effortful Control, Vineland Internalizing and Externalizing) as auxiliary variables in the imputation models. These additional auxiliary variables were not associated with retention (all *p* <.11) but were, as expected, associated with a mid-childhood autism diagnosis (*r* ~.20 to ~.50) and were not strongly associated with missingness (most *r* <.20). This addition of these auxiliary variables did not affect the pattern of findings and overall model statistics and the pattern of significant predictors was identical to that from the main models.

**Retention to 3 years**

Retention from recruitment to 3 years was 226/240 (94.2%). In univariate tests retention was higher in Phase 1 (98.0%) and Phase 2 (97.4%) than in Phase 3 (86.5%) (𝜒^2^(1, *N* = 124) = 4.89, *p* < .05 and 𝜒^2^(1, *N* = 190) = 8.46, *p* < .01, respectively) and lower in Asian/Black/Mixed compared to White children (88.5% vs. 97.8%), 𝜒^2^(1, *N* = 232) = 8.49, *p* < .01. Retention was not associated with infant sex, family income, maternal education, maternal or paternal age, proband sex or SCQ score or multiplex status (See Table S9). In the imputed multivariate logistic regression only ethnicity was associated with retention being lower in Asian/Black/Mixed than White children (*t* = 2.20, *p* < .05; all other *p* > .12).

**Three Year Recurrence and Predictors of Recurrence**

At the 3-year visit, 38 (30 boys, 8 girls: sex ratio 3.75:1) of the 226 family history siblings met DSM-5 criteria for autism. This is a sibling recurrence rate of 16.8%, 95% CIs [12.5%, 22.3%]. In univariate analysis recurrence was higher in males: 26.1%, 95% CIs [18.8%, 34.9%] than females 7.2%, 95% CIs [3.6%, 13.8%], 𝜒^2^(1, *N* = 226) = 14.39, *p* < .001, and non-significantly higher in Phase 1 (26.5%, 95% CIs [16.0%, 40.6%]) than Phase 2 (15.0%, 95% CIs [9.5%, 22.9%], 𝜒^2^(1, *N* = 162) = 3.00, *p* = .08, and Phase 3 (12.5%, 95% CIs [6.4%, 23.1%], 𝜒^2^(1, *N* = 113) = 3.61, *p* = .06) but did not differ by child ethnicity, proband sex or SCQ score, multiplex status, sibling age and maternal or paternal age. 3-year autism diagnosis was associated with lower maternal education, 𝜒^2^(1, *N* = 217) = 13.03, *p* < .01 and lower family income, 𝜒^2^(1, *N* = 209) = 10.14, *p* < .05. In the multivariate regression model sibling sex (*t* = 3.80, *p* < .001, OR = 6.00, 95% CI [2.38, 15.14]) and maternal education (*t* = 2.08, *p* < .05) remained significant and recurrence was higher in Phase 1 than Phase 3 (*t* = 1.97, *p* < .05), see Table S9.

**References**

Cornish, R. P., Macleod, J., Carpenter, J. R., & Tilling, K. (2017). Multiple imputation using linked proxy outcome data resulted in important bias reduction and efficiency gains: A simulation study. *Emerging Themes in Epidemiology*, *14*(1), 14. <https://doi.org/10.1186/s12982-017-0068-0>

Lee, K. J., Roberts, G., Doyle, L. W., Anderson, P. J., & Carlin, J. B. (2016). Multiple imputation for missing data in a longitudinal cohort study: A tutorial based on a detailed case study involving imputation of missing outcome data. *International Journal of Social Research Methodology*, *19*(5), 575–591. <https://doi.org/10.1080/13645579.2015.1126486>

| **Table S1**  *Mid-Childhood Diagnostic Outcome Child and Family Characteristics* | | | | | | | |
| --- | --- | --- | --- | --- | --- | --- | --- |
|  | Autism outcome in Mid-Childhood | | | | | |  |
|  | Autism (N=59) | | | Not autism (N=100) | | |  |
|  | *N* | *%* | | *N* | *%* | | *χ^2^ (p)* |
| Child Sex |  |  | |  |  | | 3.04 (.081) |
| Male | 35 | 59% | | 45 | 45% | |  |
| Female | 24 | 41% | | 55 | 55% | |  |
| Phase |  |  | |  |  | | 1.88 (.390) |
| 1 | 13 | 22% | | 27 | 27% | |  |
| 2 | 32 | 54% | | 43 | 43% | |  |
| 3 | 14 | 24% | | 30 | 30% | |  |
| Child Ethnicity |  |  | |  |  | | 0.87 (.352) |
| Asian/African/Black/African Caribbean/Mixed | 12 | 20% | | 14 | 15% | |  |
| White/European/Irish | 47 | 80% | | 82 | 85% | |  |
| Annual Household Income |  |  | |  |  | | 5.84 (.212) |
| Up to £20,000 | 4 | 7% | | 6 | 6% | |  |
| £20,000 to £40,000 | 18 | 32% | | 24 | 26% | |  |
| £40,000 to £60,000 | 16 | 29% | | 24 | 26% | |  |
| £60,000 to £80,000 | 11 | 19% | | 12 | 13% | |  |
| Above £80,000 | 7 | 13% | | 27 | 29% | |  |
| Maternal Highest Education |  |  | |  |  | | 3.03 (.388) |
| Up to 16/GCSE | 6 | 11% | | 8 | 8% | |  |
| Up to 18/School/College | 20 | 35% | | 24 | 25% | |  |
| Degree level | 17 | 30% | | 41 | 42% | |  |
| Postgraduate/Professional | 14 | 25% | | 24 | 25% | |  |
| Simplex vs Multiplex Status |  |  | |  |  | | 0.78 (.378) |
| Simplex | 43 | 73% | | 79 | 79% | |  |
| Multiplex | 16 | 27% | | 21 | 21% | |  |
| Proband Sex |  |  | |  |  | | 0.93 (.335) |
| Male | 50 | 85% | | 89 | 90% | |  |
| Female | 9 | 15% | | 10 | 10% | |  |
|  | *M* | *SD* | *N* | *M* | *SD* | *N* | *t-test (p)* |
| Proband SCQ score | 24.40 | (6.73) | 57 | 23.06 | (7.76) | 88 | -1.07 (.285) |
|  |  |  |  |  |  |  |  |
| Child Age (months) | 109.24 | (15.50) | 59 | 106.54 | (15.05) | 100 | -1.08 (.282) |

SCQ = Social Communication Questionnaire

| **Table S2**  *9 Month and 3 Year scores of those Seen vs. Not Seen at Mid-Childhood Visit* | | | | | | | |  |
| --- | --- | --- | --- | --- | --- | --- | --- | --- |
| Seen in Mid-Childhood  Seen MC Not seen MC | | | | | | | | |
|  | *M* | *(SD)* | *N* | *M* | *(SD)* | *N* | *t-test (p)* |  |
| **9 Months**  Mullen ELC | 99.93 | (15.12) | 162 | 97.72 | (18.40) | 74 | -0.97 (0.332) |  |
| Vineland ABC | 92.60 | (13.89) | 156 | 93.62 | (11.26) | 68 | 0.53 (0.596) |  |
| IBQ Surgency | 4.70 | (0.70) | 157 | 4.78 | (0.61) | 65 | 0.85 (0.397) |  |
| IBQ Negative Affect | 3.60 | (0.86) | 156 | 3.66 | (0.97) | 63 | 0.51 (0.614) |  |
| IBQ Effortful Control | 4.69 | (0.64) | 157 | 4.77 | (0.68) | 64 | 0.82 (0.412) |  |
| **3 Years**  Mullen ELC | 105.25 | (22.12) | 161 | 105.48 | (25.54) | 58 | 0.07 (0.947) |  |
| Vineland ABC | 95.48 | (12.67) | 159 | 94.34 | (13.53) | 56 | -0.57 (0.571) |  |
| ADOS-2 Total CSS | 2.61 | (2.28) | 163 | 2.89 | (2.48) | 61 | 0.79 (0.428) |  |
| ADI-R Toddler Algorithm | 5.18 | (7.10) | 163 | 4.26 | (6.01) | 61 | -0.89 (0.372) |  |
| SCQ Total | 6.11 | (6.91) | 154 | 6.69 | (6.39) | 59 | 0.56 (0.579) |  |
| SRS T score | 49.27 | (12.55) | 148 | 50.11 | (11.61) | 56 | 0.46 (0.649) |  |
| CBQ Surgency | 4.52 | (0.80) | 150 | 4.64 | (0.73) | 58 | 1.01 (0.313) |  |
| CBQ Negative Affect | 4.11 | (0.82) | 150 | 4.15 | (0.80) | 58 | 0.31 (0.760) |  |
| CBQ Effortful Control | 4.86 | (0.74) | 150 | 4.91 | (0.74) | 57 | 0.36 (0.716) |  |
| Vineland Internalizing (raw) | 1.99 | (2.85) | 154 | 1.45 | (1.98) | 56 | -1.33 (0.186) |  |
| Vineland Externalizing (raw) | 2.34 | (2.98) | 154 | 1.66 | (2.20) | 56 | -1.55 (0.122) |  |
| *N (%) N (%)* *χ^2^ (p)*  Autism Diagnosis at 3 Years 31 (81.6%) 7 (18.4%) 2.03 (0.154)  No Autism Diagnosis at 3 Years 132 (70.2%) 56 (29.8%) | | | | | | | |  |
|  |  |  |  |  |  |  |  |  |

ELC = Mullen Early Learning Composite, ABC = Vineland Adaptive Behavior Composite, IBQ = Infant Behavior Questionnaire, ADOS-2 CSS = Autism Diagnostic Observation Schedule-2 Calibrated Severity Score, ADI-R = Autism Diagnostic Interview-Revised, SCQ = Social Communication Questionnaire, SRS = Social Responsiveness Scale, CBQ = Child Behavior Questionnaire

| **Table S3**  *Family Characteristics and Measures of Symptoms at 3 Years by Diagnostic Outcome at 3 Years* | | | | | | | |
| --- | --- | --- | --- | --- | --- | --- | --- |
|  | Autism outcome at 36 months | | | | | | |
|  | Autism (*N*=38) | | | Not autism (*N*=188) | | |  |
|  | Count | % |  | Count | % |  | *χ^2^ (p)* |
| **Family Characteristics** |  |  |  |  |  |  |  |
| Child Sex |  |  |  |  |  |  | 14.39 (<.001) |
| Male | 30 | 79% |  | 85 | 45% |  |  |
| Female | 8 | 21% |  | 103 | 55% |  |  |
| Phase |  |  |  |  |  |  | 4.41 (.110) |
| 1 | 13 | 34% |  | 36 | 19% |  |  |
| 2 | 17 | 45% |  | 96 | 51% |  |  |
| 3 | 8 | 21% |  | 56 | 30% |  |  |
| Child Ethnicity |  |  |  |  |  |  | 0.15 (.701) |
| Asian/African/Black/African Caribbean/Mixed, etc | 7 | 18% |  | 39 | 21% |  |  |
| White/European/Irish etc | 31 | 82% |  | 145 | 79% |  |  |
| Annual Household Income |  |  |  |  |  |  | 10.14 (.038) |
| up to £20,000 | 3 | 9% |  | 16 | 9% |  |  |
| £20,000 to £40,000 | 13 | 37% |  | 49 | 28% |  |  |
| £40,000 to £60,000 | 14 | 40% |  | 42 | 24% |  |  |
| £60,000 to £80,000 | 4 | 11% |  | 25 | 14% |  |  |
| above £80,000 | 1 | 3% |  | 42 | 24% |  |  |
| Maternal Highest Education |  |  |  |  |  |  | 13.03 (.005) |
| Up to 16/GCSE | 6 | 17% |  | 13 | 7% |  |  |
| Up to 18/School/College | 17 | 49% |  | 49 | 27% |  |  |
| Degree level | 6 | 17% |  | 73 | 40% |  |  |
| Postgraduate/Professional | 6 | 17% |  | 47 | 26% |  |  |
| Simplex vs Multiplex Status |  |  |  |  |  |  | 0 (.966) |
| Simplex | 30 | 79% |  | 149 | 79% |  |  |
| Multiplex | 8 | 21% |  | 39 | 21% |  |  |
| Proband Sex |  |  |  |  |  |  | 0.02 (.883) |
| Male | 34 | 89% |  | 164 | 89% |  |  |
| Female | 4 | 11% |  | 21 | 11% |  |  |
|  | *M* | *(SD)* | *n* | *M* | *(SD)* | *n* | *t (p)* |
| Proband SCQ | 24.84 | (5.97) | 37 | 23.18 | (7.30) | 169 | -1.29 (0.200) |
|  |  |  |  |  |  |  |  |
| **3-Year scores** |  |  |  |  |  |  |  |
| Mullen ELC | 87.77 | (26.09) | 35 | 108.65 | (20.85) | 184 | 5.20 (<.001) |
| Vineland ABC | 82.11 | (13.87) | 35 | 97.66 | (11.04) | 179 | 7.29 (<.001) |
| ADOS-2 Total CSS 36 | 4.54 | (3.21) | 37 | 2.32 | (1.92) | 187 | -5.67 (<.001) |
| ADI-R Toddler Algorithm | 16.00 | (7.24) | 37 | 2.74 | (4.04) | 187 | -15.65 (<.001) |
| SCQ Total 36 | 14.56 | (7.26) | 35 | 4.58 | (5.26) | 176 | -9.57 (<.001) |
| SRS T score | 64.65 | (14.65) | 34 | 46.34 | (8.92) | 168 | -9.60 (<.001) |

ELC = Mullen Early Learning Composite, ABC = Vineland Adaptive Behavior Composite, ADOS-2 CSS = Autism Diagnostic Observation Schedule-2 Calibrated Severity Score, ADI-R = Autism Diagnostic Interview-Revised, SCQ = Social Communication Questionnaire, SRS = Social Responsiveness Scale

| **Table S4**  *Sample Characteristics of those Seen vs. Not Seen at 3 Year Visit* | | | | | | | |
| --- | --- | --- | --- | --- | --- | --- | --- |
|  | Seen at 36 months | | | | | |  |
|  | Seen 36m (*N*=246) | | | Not seen 36m (*N*=22) | | |  |
|  | *Count* | *%* |  | *Count* | *%* |  | *χ^2^ (p)* |
| Phase |  |  |  |  |  |  | 11.51 (.003) |
| 1 | 49 | 22% |  | 1 | 7% |  |  |
| 2 | 113 | 50% |  | 3 | 21% |  |  |
| 3 | 64 | 28% |  | 10 | 71% |  |  |
| Child Sex |  |  |  |  |  |  | 2.23 (.135) |
| Male | 115 | 51% |  | 10 | 71% |  |  |
| Female | 111 | 49% |  | 4 | 29% |  |  |
| Child Ethnicity |  |  |  |  |  |  | 8.49 (.004) |
| Asian/African/Black/African  Caribbean/Mixed | 46 | 21% |  | 6 | 60% |  |  |
| White/European/Irish | 176 | 79% |  | 4 | 40% |  |  |
| Annual Household Income |  |  |  |  |  |  | 3.06 (.548) |
| up to £20,000 | 19 | 9% |  | 0 | 0% |  |  |
| £20,000 to £40,000 | 62 | 30% |  | 2 | 50% |  |  |
| £40,000 to £60,000 | 56 | 27% |  | 2 | 50% |  |  |
| £60,000 to £80,000 | 29 | 14% |  | 0 | 0% |  |  |
| above £80,000 | 43 | 21% |  | 0 | 0% |  |  |
| Maternal Highest Education |  |  |  |  |  |  | 3.53 (.317) |
| Up to 16/GCSE | 19 | 9% |  | 0 | 0% |  |  |
| Up to 18/School/College | 66 | 30% |  | 1 | 12% |  |  |
| Degree level | 79 | 36% |  | 3 | 38% |  |  |
| Postgraduate/Professional | 53 | 24% |  | 4 | 50% |  |  |
| Simplex vs Multiplex Status |  |  |  |  |  |  | 0.34 (.558) |
| Simplex | 179 | 79% |  | 12 | 86% |  |  |
| Multiplex | 47 | 21% |  | 2 | 14% |  |  |
| Proband Sex |  |  |  |  |  |  | 0.12 (.725) |
| Male | 198 | 89% |  | 12 | 86% |  |  |
| Female | 25 | 11% |  | 2 | 14% |  |  |
|  | *M* | *(SD)* | *N* | *M* | *(SD)* | *N* | *t-test (p)* |
| Proband SCQ | 23.48 | (7.09) | 206 | 22.25 | (8.22) | 4 | -0.34 (0.732) |
| Mullen ELC 9m | 99.87 | (16.00) | 224 | 87.42 | (16.31) | 12 | -2.62 (0.009) |
| Vineland ABC 9m | 92.85 | (13.29) | 215 | 94.44 | (8.55) | 9 | 0.36 (0.721) |
| IBQ Surgency 9m | 4.72 | (0.67) | 212 | 4.88 | (0.71) | 10 | 0.76 (0.448) |
| IBQ Negative Affect 9m | 3.59 | (0.88) | 210 | 4.23 | (0.86) | 9 | 2.13 (0.034) |
| IBQ Effortful Control 9m | 4.71 | (0.66) | 211 | 4.75 | (0.60) | 10 | 0.19 (0.848) |

MC = Mid-childhood, SCQ = Social Communication Questionnaire, ELC = Mullen Early Learning Composite, ABC = Vineland Adaptive Behavior Composite, IBQ = Infant Behavior Questionnaire

| **Table S5**  *Family Characteristics of Earlier vs. Later Diagnosed Children.* | | | | | | | |
| --- | --- | --- | --- | --- | --- | --- | --- |
|  | Early (36m) versus late (MC) diagnosis | | | | | | |
|  | Earlier Diagnosed (*N*=28) | | | Later Diagnosed (*N*=31) | | |  |
|  | Count | % |  | Count | % | Row % | χ^2^ (p) |
| Phase |  |  |  |  |  |  | 2.83 (.243) |
| 1 | 8 | 29% |  | 5 | 16% | 38% |  |
| 2 | 12 | 43% |  | 20 | 65% | 63% |  |
| 3 | 8 | 29% |  | 6 | 19% | 43% |  |
| Child Sex |  |  |  |  |  |  | 3.24 (.072) |
| Male | 20 | 71% |  | 15 | 48% | 43% |  |
| Female | 8 | 29% |  | 16 | 52% | 67% |  |
| Child Ethnicity |  |  |  |  |  |  | 0.04 (.843) |
| Asian/African/Black/African Caribbean/Mixed, etc | 6 | 21% |  | 6 | 19% |  |  |
| White/European/Irish etc | 22 | 79% |  | 25 | 81% |  |  |
| Annual Household Income |  |  |  |  |  |  | 5.38 (.250) |
| up to £20,000 | 3 | 12% |  | 1 | 3% |  |  |
| £20,000 to £40,000 | 9 | 35% |  | 9 | 30% |  |  |
| £40,000 to £60,000 | 9 | 35% |  | 7 | 23% |  |  |
| £60,000 to £80,000 | 4 | 15% |  | 7 | 23% |  |  |
| above £80,000 | 1 | 4% |  | 6 | 20% |  |  |
| Maternal Highest Education |  |  |  |  |  |  | 6.13 (.106) |
| Up to 16/GCSE | 4 | 15% |  | 2 | 6% |  |  |
| Up to 18/School/College | 12 | 46% |  | 8 | 26% |  |  |
| Degree level | 4 | 15% |  | 13 | 42% |  |  |
| Postgraduate/Professional | 6 | 23% |  | 8 | 26% |  |  |
| Simplex vs Multiplex Status |  |  |  |  |  |  | 0.12 (.728) |
| Simplex | 21 | 75% |  | 22 | 71% |  |  |
| Multiplex | 7 | 25% |  | 9 | 29% |  |  |
| Proband Sex |  |  |  |  |  |  | 0.04 (.844) |
| Male | 24 | 86% |  | 26 | 84% |  |  |
| Female | 4 | 14% |  | 5 | 16% |  |  |
|  | *M* | *SD* | *N* | *M* | *SD* | *N* | *t-test (p)* |
| Proband SCQ | 25.30 | (5.93) | 27 | 23.60 | (7.39) | 30 | 0.95 (.347) |

SCQ = Social Communication Questionnaire

| **Table S6**  *Number of Children Meeting Cut-off Criteria on Autism Diagnostic Measures* | | | | |
| --- | --- | --- | --- | --- |
|  | Autism outcome in Mid-Childhood | | | |
|  | Autism (*N*=59) | | Not autism (*N*=99) | |
|  | Count | % | Count | % |
| ADOS-2^1^ |  |  |  |  |
| Non-Spectrum | 21 | 36% | 82 | 84% |
| Autism Spectrum | 9 | 15% | 8 | 8% |
| Autism | 29 | 49% | 8 | 8% |
| ADI-R^2^ |  |  |  |  |
| Non-Spectrum | 16 | 28% | 94 | 95% |
| Autism Spectrum | 13 | 22% | 4 | 4% |
| Autism | 29 | 50% | 1 | 1% |
| Either/Both ADOS-2 or ADI-R^3^ |  |  |  |  |
| Non-Spectrum | 6 | 10% | 78 | 80% |
| Autism Spectrum | 8 | 14% | 10 | 10% |
| Autism | 44 | 76% | 9 | 9% |

^1^ N=59 and 98; ^2^ N=58 and 99; ^3^ N=58 and 97. ADOS-2 = Autism Diagnostic Observation Schedule-2, ADI-R = Autism Diagnostic Interview-Revised. Note: We used the modified Risi et al. (2006) ADI-R criteria for broader autism spectrum disorder (ASD)

**Table S7**

*Characteristics at 3 Years of the Later Diagnosed Children*

|  |  | **ADOS** | **ADI** | | | | |  |  |  |  |
| --- | --- | --- | --- | --- | --- | --- | --- | --- | --- | --- | --- |
| **Child** | **Sex** | **CSS** | **SOC** | **COM** | **RRB** | **ONSET** | **Toddler** | **SCQ** | **Parental concerns** | **Researcher concerns** | **Certainty** |
| 1 | M | 1 | 0 | 1 | 1 | 0 | 1 | 3 | No | No | High |
| 2 | M | 8 | 4 | 6 | 0 | 2 | 8 | 4 | Language | Attention | Low |
| 3 | M | 1 | 0 | 0 | 0 | 0 | 0 | 1 | No | RRB | High |
| 4 | M | 3 | 10 | 9 | 7 | 3 | 14 | 16 | Language, RRB, Play | RRB | Low |
| 5 | F | 4 | 0 | 4 | 0 | 1 | 0 |  | Language | Language, Developmental delay, Activity level | High |
| 6 | F | 1 | 2 | 4 | 1 | 0 | 2 | 3 | Language | Language | High |
| 7 | F | 1 | 5 | 1 | 0 | 0 | 4 | 1 | No | No | High |
| 8 | M | 3 | 4 | 11 | 3 | 2 | 7 | 12 | Behaviour | Activity level | Low |
| 9 | F | 1 | 11 | 5 | 1 | 0 | 9 | 23 | Behaviour, Social interaction, Sensory | No | Med |
| 10 | M | 2 | 0 | 3 | 2 | 0 | 5 | 4 | Sensory | RRB, attention | High |
| 11 | F | 1 | 3 | 2 | 1 | 0 | 2 | 5 | Sleep and developmental (at 24m - not 36m) | No | High |
| 12 | F | 1 | 2 | 4 | 0 | 2 | 3 | 8 | Language | RRB, social engagement | Med |
| 13 | M | 1 | 0 | 1 | 0 | 0 | 0 | 4 | No | No | High |
| 14 | M | 6 | 2 | 1 | 0 | 0 | 2 | 2 | Language, Social interaction (as baby - not 36m) | Attention, Processing speed | High |
| 15 | F | 7 | 3 | 0 | 0 | 0 | 3 | 1 | No | Social engagement, Sensory | Med |
| 16 | F | 1 | 1 | 1 | 0 | 0 | 1 | 3 | No | No | High |
| 17 | F | 4 | 1 | 2 | 2 | 1 | 4 | 6 | Language, ASD traits (at 24m - not 36m) | Social engagement | Med |
| 18 | F | 2 | 0 | 1 | 0 | 0 | 0 | 1 | No | Social engagement |  |
| 19 | M | 1 | 3 | 11 | 1 | 1 | 2 | 4 | Language, ASD traits | Language, Activity level | High |
| 20 | M | 1 | 1 | 0 | 0 | 0 | 4 | 9 | Language, ASD traits | Language, RRB | High |
| 21 | F | 3 | 3 | 4 | 2 | 2 | 5 | 15 | Social Interaction, Language | RRB | Med |
| 22 | M | 7 | 0 | 1 | 1 | 0 | 0 | 5 | RRB | RRB | Med |
| 23 | F | 5 | 0 | 1 | 1 | 0 | 0 | 2 | Sensory | RRB, Play | Med |
| 24 | M | 2 | 0 | 0 | 0 | 0 | 0 | 4 | No | No | Med |
| 25 | F | 1 | 4 | 3 | 0 | 0 | 4 | 5 | No | No | Med |
| 26 | F | 3 | 0 | 1 | 0 | 3 | 0 | 3 | Language | Language | High |
| 27 | M | 1 | 4 | 9 | 2 | 2 | 8 | 3 | Language | Language, Sensory, RRB | Med |
| 28 | F | 1 | 7 | 2 | 4 | 3 | 12 |  | Language | Language, Sensory, RRB | Low |
| 29 | F | 1 | 1 | 0 | 1 | 0 | 2 | 5 | PDA | No | High |
| 30 | M | 2 | 2 | 1 | 1 | 0 | 2 | 3 | OCD | Social interaction, RRB | High |
| 31 | M | 1 | 5 | 4 | 1 | 3 | 5 | 2 | Language | Language, activity level | High |

ADOS-2 CSS = Autism Diagnostic Observation Schedule-2 Calibrated Severity Score, ADI-R = Autism Diagnostic Interview-Revised, SOC = ADI Social domain, COM = ADI Communication domain, RRB = ADI Restricted and Repetitive Behaviours domain, ONSET = ADI Onset domain, Toddler = ADI Toddler Algorithm score, SCQ = Social Communication Questionnaire

| **Table S8**  *Background Characteristics of Those With and Without a Community Diagnosis of Autism* | | | | | | | | | | | | | | |
| --- | --- | --- | --- | --- | --- | --- | --- | --- | --- | --- | --- | --- | --- | --- |
|  | Community vs. No Community Diagnosis | | | | | | | Research and Community Diagnosis vs. Research Diagnosis Only | | | | | | |
|  | Community Diagnosis (N=39) | | | No Community Diagnosis (N=120) | | |  | Research and Community (N=37) | | | Research only (N=22) | | |  |
|  | N | % | Row % | N | % | Row % | *χ^2^ (p)* | N | % | Row % | N | % | Row % | *χ^2^ (p)* |
| Phase |  |  |  |  |  |  |  |  |  |  |  |  |  | 2.10 (.351) |
| 1 | 7 | 18% | 18% | 33 | 28% | 83% | 2.08 (.353) | 6 | 16% | 46% | 7 | 32% | 54% |  |
| 2 | 22 | 56% | 29% | 53 | 44% | 71% |  | 21 | 57% | 66% | 11 | 50% | 34% |  |
| 3 | 10 | 26% | 23% | 34 | 28% | 77% |  | 10 | 27% | 71% | 4 | 18% | 29% |  |
| Child Sex |  |  |  |  |  |  | 2.60 (.107) |  |  |  |  |  |  | 0.33 (.565) |
| Male | 24 | 62% | 30% | 56 | 47% | 70% |  | 23 | 62% | 66% | 12 | 55% | 34% |  |
| Female | 15 | 38% | 19% | 64 | 53% | 81% |  | 14 | 38% | 58% | 10 | 45% | 42% |  |
| Child Ethnicity |  |  |  |  |  |  | 1.48 (.223) |  |  |  |  |  |  | 0.97 (.324) |
| Asian/African/Black/African Caribbean/Mixed, etc | 9 | 23% | 35% | 17 | 15% | 65% |  | 9 | 24% | 75% | 3 | 14% | 25% |  |
| White/European/Irish etc | 30 | 77% | 23% | 99 | 85% | 77% |  | 28 | 76% | 60% | 19 | 86% | 40% |  |
| Annual Household Income |  |  |  |  |  |  | 4.73 (.316) |  |  |  |  |  |  | 3.72 (.455) |
| up to £20,000 | 4 | 11% | 40% | 6 | 5% | 60% |  | 4 | 12% | 100% | 0 | 0% | 0% |  |
| £20,000 to £40,000 | 12 | 33% | 29% | 30 | 27% | 71% |  | 11 | 32% | 61% | 7 | 32% | 39% |  |
| £40,000 to £60,000 | 10 | 28% | 25% | 30 | 27% | 75% |  | 10 | 29% | 63% | 6 | 27% | 38% |  |
| £60,000 to £80,000 | 6 | 17% | 26% | 17 | 15% | 74% |  | 6 | 18% | 55% | 5 | 23% | 45% |  |
| above £80,000 | 4 | 11% | 12% | 30 | 27% | 88% |  | 3 | 9% | 43% | 4 | 18% | 57% |  |
| Maternal Highest Education |  |  |  |  |  |  | 2.36 (.501) |  |  |  |  |  |  | 1.20 (.752) |
| Up to 16/GCSE | 4 | 11% | 29% | 10 | 9% | 71% |  | 4 | 11% | 67% | 2 | 10% | 33% |  |
| Up to 18/School/College | 14 | 37% | 32% | 30 | 26% | 68% |  | 14 | 39% | 70% | 6 | 29% | 30% |  |
| Degree level | 11 | 29% | 19% | 47 | 41% | 81% |  | 9 | 25% | 53% | 8 | 38% | 47% |  |
| Postgraduate/Professional | 9 | 24% | 24% | 29 | 25% | 76% |  | 9 | 25% | 64% | 5 | 24% | 36% |  |
| Simplex vs Multiplex Status |  |  |  |  |  |  | 2.93 (.087) |  |  |  |  |  |  | 1.42 (.234) |
| Simplex | 26 | 67% | 21% | 96 | 80% | 79% |  | 25 | 68% | 58% | 18 | 82% | 42% |  |
| Multiplex | 13 | 33% | 35% | 24 | 20% | 65% |  | 12 | 32% | 75% | 4 | 18% | 25% |  |
| Proband Sex |  |  |  |  |  |  | 0.03 (.860) |  |  |  |  |  |  | 0.64 (.424) |
| Male | 34 | 87% | 24% | 105 | 88% | 76% |  | 33 | 89% | 65% | 18 | 82% | 35% |  |
| Female | 5 | 13% | 26% | 14 | 12% | 74% |  | 4 | 11% | 50% | 4 | 18% | 50% |  |
|  | *M* | *(SD)* | *N* | *M* | *(SD)* | *N* | *t-test (p)* | *M* | *(SD)* | *N* | *M* | *(SD)* | *N* | *t-test (p)* |
| Proband SCQ | 23.92 | (7.30) | 38 | 23.47 | (7.44) | 107 | -.32 (.746) | 23.97 | (7.40) | 36 | 25.14 | (5.49) | 21 | 0.63 (.532) |

SCQ = Social Communication Questionnaire

| **Table S9: Multilevel Logistic Regression Model Summaries (imputed values)** |
| --- |

| *Table S9.1 Retention from Recruitment to Mid-Childhood*   \|  \| *Coefficient* \| *SE* \| *t* \| *p* \| *[95% conf. interval]* \| \| \| --- \| --- \| --- \| --- \| --- \| --- \| --- \| \|  \|  \|  \|  \|  \|  \|  \| \| Sibling sex \| -.19 \| .31 \| -0.60 \| 0.547 \| -.79 \| .42 \| \|  \|  \|  \|  \|  \|  \|  \| \| Phase (1) \|  \|  \|  \|  \|  \|  \| \| 2 \| -.58 \| .45 \| -1.29 \| 0.196 \| -1.46 \| .30 \| \| **3** \| **-1.13** \| **.47** \| **-2.41** \| **0.016** \| **-2.05** \| **-.21** \| \|  \|  \|  \|  \|  \|  \|  \| \| **Simplex/Multiplex** \|  \|  \|  \|  \|  \|  \| \|  \| **.87** \| **.42** \| **2.10** \| **0.035** \| **.06** \| **1.69** \| \| Proband SCQ \| .01 \| .02 \| 0.58 \| 0.563 \| -.03 \| .06 \| \| Proband sex \| -.04 \| .49 \| -0.08 \| 0.933 \| -1.00 \| .92 \| \|  \|  \|  \|  \|  \|  \|  \| \| **Ethnicity** \|  \|  \|  \|  \|  \|  \| \|  \| **1.00** \| **.36** \| **2.82** \| **0.005** \| **.31** \| **1.70** \| \| Maternal education \| -.18 \| .19 \| -0.94 \| 0.350 \| -.55 \| .20 \| \| Family income \| .25 \| .15 \| 1.63 \| 0.102 \| -.05 \| .54 \| \| Maternal age \| .08 \| .05 \| 1.79 \| 0.074 \| -.01 \| .17 \| \| Paternal age \| -.01 \| .03 \| -0.33 \| 0.743 \| -.08 \| .06 \| \| Constant \| -2.39 \| 1.54 \| -1.56 \| 0.119 \| -5.41 \| .62 \| \|  \| *N* = 240 (imputed) *F* = 2.24, *p* = .010 \| \| \| \| \| \|   SCQ = Social Communication Questionnaire |
| --- | --- | --- | --- | --- | --- | --- | --- | --- | --- | --- | --- | --- | --- | --- | --- | --- | --- | --- | --- | --- | --- | --- | --- | --- | --- | --- | --- | --- | --- | --- | --- | --- | --- | --- | --- | --- | --- | --- | --- | --- | --- | --- | --- | --- | --- | --- | --- | --- | --- | --- | --- | --- | --- | --- | --- | --- | --- | --- | --- | --- | --- | --- | --- | --- | --- | --- | --- | --- | --- | --- | --- | --- | --- | --- | --- | --- | --- | --- | --- | --- | --- | --- | --- | --- | --- | --- | --- | --- | --- | --- | --- | --- | --- | --- | --- | --- | --- | --- | --- | --- | --- | --- | --- | --- | --- | --- | --- | --- | --- | --- | --- | --- | --- | --- | --- | --- | --- | --- | --- | --- | --- | --- | --- | --- | --- | --- | --- | --- | --- | --- | --- | --- | --- | --- | --- | --- | --- | --- | --- | --- | --- | --- | --- | --- | --- | --- | --- |

| *Table S9.2 Retention from 9 months to Mid-Childhood* |
| --- |

| *9 months* | *Coefficient* | *SE* | *t* | *p* | *[95% conf. interval]* | |
| --- | --- | --- | --- | --- | --- | --- |
| Mullen ELC | .01 | .01 | 1.27 | 0.204 | -.01 | .03 |
| Vineland ABC | -.00 | .013 | -0.32 | 0.749 | -.03 | .02 |
| IBQ Surgency | -.16 | .27 | -0.57 | 0.567 | -.69 | .38 |
| IBQ Negative Affect | -.19 | .19 | -1.00 | 0.319 | -.56 | .18 |
| IBQ Effortful Control | -.22 | .28 | -0.77 | 0.444 | -.78 | .34 |
| Constant | 2.36 | 1.76 | 1.34 | 0.179 | -1.08 | 5.81 |
|  | *N* = 240 (imputed) *F* = 0.74, *p* = .590 | | | | | |

ELC = Mullen Early Learning Composite, ABC = Vineland Adaptive Behavior Composite, IBQ = Infant Behavior Questionnaire

| *Table S9.3 Retention from 3 Years to Mid-Childhood* |
| --- |

| 3 years | *Coefficient* | *SE* | *t* | *p* | *[95% conf. interval]* | |
| --- | --- | --- | --- | --- | --- | --- |
| Mullen ELC | -.012394 | .0107625 | -1.15 | 0.250 | -.0335067 | .0087188 |
| Vineland ABC | .0398073 | .0228453 | 1.74 | 0.082 | -.0050102 | .0846249 |
| ADOS-2 CSS | -.1370056 | .0769274 | -1.78 | 0.075 | -.2878458 | .0138345 |
| ADI Toddler Total | .1112551 | .0625773 | 1.78 | 0.076 | -.0115281 | .2340384 |
| SCQ | -.0508324 | .0523279 | -0.97 | 0.332 | -.1535508 | .0518859 |
| SRS-2 Total score | -.0226237 | .0145314 | -1.56 | 0.120 | -.0511356 | .0058881 |
|  |  |  |  |  |  |  |
| Autism Diagnosis |  |  |  |  |  |  |
|  | .7453906 | .699066 | 1.07 | 0.286 | -.6254123 | 2.116193 |
| CBQ Surgency | -.2676245 | .2336747 | -1.15 | 0.252 | -.7261831 | .1909341 |
| CBQ Negative Affect | -.111294 | .2527804 | -0.44 | 0.660 | -.6075896 | .3850017 |
| CBQ Effortful Control | -.1368118 | .2593589 | -0.53 | 0.598 | -.645815 | .3721914 |
| Vineland Internalizing | .1304429 | .1155384 | 1.13 | 0.259 | -.0963549 | .3572407 |
| Vineland Externalizing | .1074946 | .0844246 | 1.27 | 0.203 | -.058298 | .2732871 |
| _Constant | 1.225195 | 2.355407 | 0.52 | 0.603 | -3.395826 | 5.846216 |
|  | *N* = 240 (imputed) *F* = 1.32, *p* = .199 | | | | | |

ELC = Mullen Early Learning Composite, ABC = Vineland Adaptive Behavior Composite, ADOS-2 CSS = Autism Diagnostic Observation Schedule-2 Calibrated Severity Score, ADI-R = Autism Diagnostic Interview-Revised, SCQ = Social Communication Questionnaire, SRS = Social Responsiveness Scale CBQ = Child Behavior Questionnaire

| *Table S9.4 Mid-Childhood Diagnostic Outcome by Background Characteristics*   \|  \| *Coefficient* \| *SE* \| *t* \| *p* \| *[95% conf. interval]* \| \| \| --- \| --- \| --- \| --- \| --- \| --- \| --- \| \| **Sibling Sex** \|  \|  \|  \|  \|  \|  \| \|  \| **.73** \| **.37** \| **1.99** \| **0.049** \| **-.00** \| **1.46** \| \|  \|  \|  \|  \|  \|  \|  \| \| Phase (1) \|  \|  \|  \|  \|  \|  \| \| 2 \| .17 \| .65 \| 0.26 \| 0.794 \| -1.10 \| 1.43 \| \| 3 \| -.22 \| .57 \| 0.38 \| 0.706 \| -1.34 \| .90 \| \|  \|  \|  \|  \|  \|  \|  \| \| Multiplex/Simplex \|  \|  \|  \|  \|  \|  \| \|  \| .45 \| .44 \| 1.03 \| 0.301 \| -.40 \| 1.31 \| \| Proband SCQ \| .02 \| .026 \| 0.86 \| 0.387 \| -.03 \| .07 \| \| Proband sex \| .56 \| .53 \| 1.06 \| 0.290 \| -.48 \| 1.60 \| \|  \|  \|  \|  \|  \|  \|  \| \| Ethnicity \|  \|  \|  \|  \|  \|  \| \|  \| -.30 \| .48 \| 0.63 \| 0.529 \| -1.24 \| .63 \| \| Maternal education \| -.09 \| .21 \| 0.41 \| 0.681 \| -.50 \| .33 \| \| Family income \| -.20 \| .17 \| 1.22 \| 0.222 \| -.53 \| .12 \| \| Sibling age \| .01 \| .02 \| 0.45 \| 0.653 \| -.03 \| .04 \| \| Maternal age \| .07 \| .06 \| 1.31 \| 0.191 \| -.04 \| .18 \| \| Paternal age \| -.05 \| .04 \| 1.14 \| 0.255 \| -.13 \| .04 \| \| Constant \| -2.08 \| 2.38 \| 0.87 \| 0.383 \| -6.75 \| 2.59 \| \|  \| *N* = 159 (imputed) *F* = 0.97, *p* = .475 \| \| \| \| \| \| |
| --- | --- | --- | --- | --- | --- | --- | --- | --- | --- | --- | --- | --- | --- | --- | --- | --- | --- | --- | --- | --- | --- | --- | --- | --- | --- | --- | --- | --- | --- | --- | --- | --- | --- | --- | --- | --- | --- | --- | --- | --- | --- | --- | --- | --- | --- | --- | --- | --- | --- | --- | --- | --- | --- | --- | --- | --- | --- | --- | --- | --- | --- | --- | --- | --- | --- | --- | --- | --- | --- | --- | --- | --- | --- | --- | --- | --- | --- | --- | --- | --- | --- | --- | --- | --- | --- | --- | --- | --- | --- | --- | --- | --- | --- | --- | --- | --- | --- | --- | --- | --- | --- | --- | --- | --- | --- | --- | --- | --- | --- | --- | --- | --- | --- | --- | --- | --- | --- | --- | --- | --- | --- | --- | --- | --- | --- | --- | --- | --- | --- | --- | --- | --- | --- | --- | --- | --- | --- | --- | --- | --- | --- | --- | --- | --- | --- | --- | --- | --- | --- | --- | --- | --- | --- | --- |

SCQ = Social Communication Questionnaire

| *Table S9.5 3-Year Retention by Background Characteristics* |
| --- |

|  | *Coefficient* | *SE* | *t* | *p* | *[95% conf. interval]* | |
| --- | --- | --- | --- | --- | --- | --- |
| Sibling sex |  |  |  |  |  |  |
|  | -.60 | .82 | -0.73 | 0.468 | -2.21 | 1.02 |
|  |  |  |  |  |  |  |
| Phase (1) |  |  |  |  |  |  |
| 2 | -.39 | 1.66 | -0.23 | 0.816 | -3.64 | 2.87 |
| 3 | -2.70 | 1.71 | -1.58 | 0.114 | -6.06 | .65 |
|  |  |  |  |  |  |  |
| Multiplex/Simplex |  |  |  |  |  |  |
|  | .88 | 1.18 | 0.75 | 0.453 | -1.43 | 3.19 |
| Proband SCQ | .049 | .08 | 0.59 | 0.559 | -.12 | .21 |
| Proband sex | -.79 | 1.20 | -0.66 | 0.512 | -3.15 | 1.57 |
|  |  |  |  |  |  |  |
| Ethnicity |  |  |  |  |  |  |
|  | 1.89 | .86 | 2.20 | 0.028 | .20 | 3.58 |
| Maternal education | -1.01 | .66 | -1.54 | 0.126 | -2.31 | .29 |
| Family income | .79 | .75 | 1.06 | 0.294 | -.70 | 2.27 |
| Maternal age | -.073 | .16 | -0.45 | 0.650 | -.39 | .24 |
| Paternal age | -.02 | .12 | -0.19 | 0.848 | -.26 | .21 |
| _Constant | 6.98 | 5.24 | 1.33 | 0.183 | -3.31 | 17.27 |
|  | *N* = 240 (imputed) *F* = 0.99, *p* = .453 | | | | | |

SCQ = Social Communication Questionnaire

Table S9.6 3-Year Diagnostic Outcome by Background Characteristics

|  | *Coefficient* | *SE.* | *t* | *p* | *[95% conf. interval]* | |
| --- | --- | --- | --- | --- | --- | --- |
| **Sibling sex** |  |  |  |  |  |  |
|  | **1.79** | **.47** | **3.80** | **0.000** | **.87** | **2.72** |
|  |  |  |  |  |  |  |
| Phase (1) |  |  |  |  |  |  |
| 2 | -.94 | .50 | -1.87 | 0.061 | -1.93 | .045 |
| **3** | **-1.12** | **.56** | **-1.99** | **0.046** | **-2.23** | **-.019** |
|  |  |  |  |  |  |  |
| Multiplex/Simplex |  |  |  |  |  |  |
|  | .25 | .51 | 0.48 | 0.633 | -.76 | 1.25 |
| Proband SCQ | .014 | .031 | 0.46 | 0.647 | -.05 | .08 |
| Proband sex | -.10 | .65 | -0.16 | 0.874 | -1.37 | 1.16 |
|  |  |  |  |  |  |  |
| Ethnicity |  |  |  |  |  |  |
|  | .25 | .52 | 0.48 | 0.629 | -.77 | 1.28 |
| Maternal education | -.49 | .24 | -2.08 | 0.037 | -.96 | -.029 |
| Family income | -.370 | .21 | -1.79 | 0.073 | -.77 | .03 |
| Maternal age | -.07 | .11 | -0.58 | 0.559 | -.29 | .157 |
| Paternal age | .01 | .04 | 0.24 | 0.812 | -.065 | .082 |
| _Constant | 1.97 | 5.17 | 0.38 | 0.703 | -8.16 | 12.09 |
|  | *N* = 226 (imputed) *F* = 2.45, *p* = .005 | | | | | |

SCQ = Social Communication Questionnaire

| *Table S9.7 Early vs. Late Diagnosis by Background Characteristics*   \|  \| *Coefficient* \| *SE* \| *t* \| *p* \| *[95% conf. interval]* \| \| \| --- \| --- \| --- \| --- \| --- \| --- \| --- \| \| Sibling sex \|  \|  \|  \|  \|  \|  \| \|  \| -1.45 \| .77 \| -1.89 \| 0.059 \| -2.95 \| .054 \| \|  \|  \|  \|  \|  \|  \|  \| \| Phase (1) \|  \|  \|  \|  \|  \|  \| \| 2 \| .53 \| 1.39 \| 0.38 \| 0.703 \| -2.19 \| 3.24 \| \| 3 \| -.27 \| 1.13 \| -0.24 \| 0.809 \| -2.50 \| 1.95 \| \|  \|  \|  \|  \|  \|  \|  \| \| Multiplex/Simplex \|  \|  \|  \|  \|  \|  \| \|  \| 1.67 \| .96 \| 1.74 \| 0.081 \| -.21 \| 3.54 \| \| Proband SCQ \| -.05 \| .05 \| -0.98 \| 0.325 \| -.16 \| .05 \| \| Proband sex \| 1.05 \| .94 \| 1.11 \| 0.265 \| -.80 \| 2.90 \| \|  \|  \|  \|  \|  \|  \|  \| \| Ethnicity \|  \|  \|  \|  \|  \|  \| \|  \| .37 \| .87 \| 0.42 \| 0.674 \| -1.34 \| 2.08 \| \| Maternal education \| .34 \| .37 \| 0.92 \| 0.356 \| -.38 \| 1.07 \| \| Family income \| .53 \| .34 \| 1.58 \| 0.115 \| -.13 \| 1.20 \| \| Sibling age \| .024 \| .037 \| 0.64 \| 0.521 \| -.05 \| .10 \| \| Maternal age \| .16 \| .12 \| 1.42 \| 0.156 \| -.06 \| .39 \| \| Paternal age \| .00 \| .09 \| 0.03 \| 0.974 \| -.17 \| .17 \| \| Constant \| -9.80 \| 5.75 \| -1.71 \| 0.088 \| -21.06 \| 1.46 \| \|  \| *N* = 59 (imputed) *F* = 0.89, *p* = .567 \| \| \| \| \| \| |
| --- | --- | --- | --- | --- | --- | --- | --- | --- | --- | --- | --- | --- | --- | --- | --- | --- | --- | --- | --- | --- | --- | --- | --- | --- | --- | --- | --- | --- | --- | --- | --- | --- | --- | --- | --- | --- | --- | --- | --- | --- | --- | --- | --- | --- | --- | --- | --- | --- | --- | --- | --- | --- | --- | --- | --- | --- | --- | --- | --- | --- | --- | --- | --- | --- | --- | --- | --- | --- | --- | --- | --- | --- | --- | --- | --- | --- | --- | --- | --- | --- | --- | --- | --- | --- | --- | --- | --- | --- | --- | --- | --- | --- | --- | --- | --- | --- | --- | --- | --- | --- | --- | --- | --- | --- | --- | --- | --- | --- | --- | --- | --- | --- | --- | --- | --- | --- | --- | --- | --- | --- | --- | --- | --- | --- | --- | --- | --- | --- | --- | --- | --- | --- | --- | --- | --- | --- | --- | --- | --- | --- | --- | --- | --- | --- | --- | --- | --- | --- | --- | --- | --- | --- | --- | --- |

SCQ = Social Communication Questionnaire

| *Table S9.8 Community versus No Community Diagnosis by Background Characteristics*   \|  \| *Coefficient* \| *SE* \| *t* \| *p* \| *[95% conf. interval]* \| \| \| --- \| --- \| --- \| --- \| --- \| --- \| --- \| \| **Proband sex** \|  \|  \|  \|  \|  \|  \| \|  \| **.87** \| **.43** \| **2.02** \| **0.043** \| **.03** \| **1.72** \| \|  \|  \|  \|  \|  \|  \|  \| \| Phase (1) \|  \|  \|  \|  \|  \|  \| \| 2 \| -.05 \| .77 \| -0.06 \| 0.952 \| -1.55 \| 1.46 \| \| 3 \| -.14 \| .67 \| -0.21 \| 0.830 \| -1.45 \| 1.16 \| \|  \|  \|  \|  \|  \|  \|  \| \| Multiplex/Simplex \|  \|  \|  \|  \|  \|  \| \|  \| .82 \| .48 \| 1.73 \| 0.084 \| -.11 \| 1.76 \| \| Proband SCQ \| -.00 \| .03 \| -0.12 \| 0.908 \| -.06 \| .06 \| \| Proband sex \| .05 \| .61 \| 0.09 \| 0.930 \| -1.13 \| 1.24 \| \|  \|  \|  \|  \|  \|  \|  \| \| Ethnicity \|  \|  \|  \|  \|  \|  \| \|  \| -.37 \| .51 \| -0.73 \| 0.468 \| -1.38 \| .64 \| \| Maternal education \| -.03 \| .24 \| -0.13 \| 0.900 \| -.50 \| .44 \| \| Family income \| -.27 \| .19 \| -1.38 \| 0.167 \| -.65 \| .11 \| \| Sibling age \| .02 \| .02 \| 1.03 \| 0.305 \| -.02 \| .06 \| \| Maternal age \| .06 \| .07 \| 0.87 \| 0.386 \| -.07 \| .18 \| \| Paternal age \| -.06 \| .05 \| -1.07 \| 0.287 \| -.16 \| .05 \| \| Constant \| -2.521 \| 2.67 \| -0.94 \| 0.345 \| -7.75 \| 2.71 \| \|  \| *N* = 159 (imputed) *F* = 1.02, *p* = .426 \| \| \| \| \| \| |
| --- | --- | --- | --- | --- | --- | --- | --- | --- | --- | --- | --- | --- | --- | --- | --- | --- | --- | --- | --- | --- | --- | --- | --- | --- | --- | --- | --- | --- | --- | --- | --- | --- | --- | --- | --- | --- | --- | --- | --- | --- | --- | --- | --- | --- | --- | --- | --- | --- | --- | --- | --- | --- | --- | --- | --- | --- | --- | --- | --- | --- | --- | --- | --- | --- | --- | --- | --- | --- | --- | --- | --- | --- | --- | --- | --- | --- | --- | --- | --- | --- | --- | --- | --- | --- | --- | --- | --- | --- | --- | --- | --- | --- | --- | --- | --- | --- | --- | --- | --- | --- | --- | --- | --- | --- | --- | --- | --- | --- | --- | --- | --- | --- | --- | --- | --- | --- | --- | --- | --- | --- | --- | --- | --- | --- | --- | --- | --- | --- | --- | --- | --- | --- | --- | --- | --- | --- | --- | --- | --- | --- | --- | --- | --- | --- | --- | --- | --- | --- | --- | --- | --- | --- | --- | --- |

SCQ = Social Communication Questionnaire

*Table S9.9 Community versus Research Diagnosis by Mid-Childhood Characteristics*

|  | *Coefficient* | *SE* | *t* | *p* | *[95% conf. interval]* | |
| --- | --- | --- | --- | --- | --- | --- |
| Sibling sex |  |  |  |  |  |  |
|  | -.96 | .95 | -1.01 | 0.313 | -2.83 | .91 |
|  |  |  |  |  |  |  |
| Phase (1) |  |  |  |  |  |  |
| 2 | -1.32 | 1.85 | -0.71 | 0.477 | -4.95 | 2.31 |
| 3 | -.61 | 1.47 | -0.42 | 0.677 | -3.50 | 2.28 |
|  |  |  |  |  |  |  |
| Sibling age | .09 | .05 | 1.89 | 0.059 | -.00 | .19 |
| WASI FSIQ | -.01 | .02 | -0.42 | 0.677 | -.05 | .04 |
| Vineland ABC | .00 | .04 | 0.06 | 0.956 | -.08 | .08 |
| ADOS-2 CSS | .04 | .18 | 0.25 | 0.801 | -.31 | .40 |
| SCQ score | .20 | .09 | 2.13 | 0.034 | .02 | .38 |
| Conners-3 AN T | .03 | .05 | 0.75 | 0.455 | -.06 | .12 |
| Conners-3 AH T | .02 | .04 | 0.56 | 0.577 | -.06 | .11 |
| SCAS-P T | -.04 | .06 | -0.70 | 0.481 | -.15 | .07 |
| _Constant | -11.61 | 6.92 | -1.68 | 0.094 | -25.19 | 1.97 |
|  | *N* = 59 (imputed) *F* = 0.98, *p* = .460 | | | | | |

WASI = Wechsler Abbreviated Scale of Intelligence; FSIQ = Full Scale IQ; ABC = Vineland Adaptive Behavior Composite, ADOS-2 CSS = Autism Diagnostic Observation Schedule-2 Calibrated Severity Score, SCQ = Social Communication Questionnaire, AN = Inattentive, AH = Hyperactivity, SCAS-P = Spence Children’s Anxiety Scale - Parent, T = t-scores.

*Table S9.10 Community versus Research Diagnosis by 3-Year Characteristics*

|  | *Coefficient* | *SE.* | *t* | *p* | *[95% conf. interval]* | |
| --- | --- | --- | --- | --- | --- | --- |
| Sibling sex |  |  |  |  |  |  |
|  | -.57 | .81 | -0.70 | 0.484 | -2.16 | 1.02 |
|  |  |  |  |  |  |  |
| Phase (1) |  |  |  |  |  |  |
| 2 | .25 | 1.58 | 0.16 | 0.877 | -2.86 | 3.35 |
| 3 | .89 | 1.44 | 0.62 | 0.534 | -1.92 | 3.70 |
|  |  |  |  |  |  |  |
| Sibling age | .023 | .04 | 0.59 | 0.556 | -.06 | .11 |
| Mullen ELC | -.02 | .02 | -0.95 | 0.343 | -.07 | .02 |
| Vineland ABC | -.01 | .05 | -0.18 | 0.860 | -.10 | .08 |
| ADOS-2 CSS | .06 | .16 | 0.37 | 0.715 | -.26 | .38 |
| ADI Toddler Total | .01 | .10 | 0.09 | 0.931 | -.19 | .20 |
| SCQ score | .19 | .10 | 1.84 | 0.066 | -.01 | .40 |
| Vineland Internalizing | -.10 | .17 | -0.59 | 0.552 | -.44 | .23 |
| Vineland Externalizing | -.12 | .13 | -0.92 | 0.359 | -.37 | .14 |
| Constant | -.52 | 5.22 | -0.10 | 0.921 | -10.75 | 9.72 |
|  | *N* = 59 (imputed) *F* = 0.98, *p* = .459 | | | | | |

ELC = Mullen Early Learning Composite, ABC = Vineland Adaptive Behavior Composite, ADOS-2 CSS = Autism Diagnostic Observation Schedule-2 Calibrated Severity Score, SCQ = Social Communication Questionnaire.
